# Transport of Magnesium by a Bacterial Nramp-Related Gene

**DOI:** 10.1371/journal.pgen.1004429

**Published:** 2014-06-26

**Authors:** Jung-Ho Shin, Catherine A. Wakeman, Jonathan R. Goodson, Dmitry A. Rodionov, Benjamin G. Freedman, Ryan S. Senger, Wade C. Winkler

**Affiliations:** 1 The University of Maryland, Department of Cell Biology and Molecular Genetics, College Park, Maryland, United States of America; 2 The University of Texas Southwestern Medical Center, Department of Biochemistry, Dallas, Texas, United States of America; 3 Sanford-Burnham Medical Research Institute, La Jolla, California, United States of America; 4 A.A.Kharkevich Institute for Information Transmission Problems, Russian Academy of Sciences, Moscow, Russia; 5 Virginia Tech University, Department of Biological Systems Engineering, Blacksburg, Virginia, United States of America; Universidad de Sevilla, Spain

## Abstract

Magnesium is an essential divalent metal that serves many cellular functions. While most divalent cations are maintained at relatively low intracellular concentrations, magnesium is maintained at a higher level (∼0.5–2.0 mM). Three families of transport proteins were previously identified for magnesium import: CorA, MgtE, and MgtA/MgtB P-type ATPases. In the current study, we find that expression of a bacterial protein unrelated to these transporters can fully restore growth to a bacterial mutant that lacks known magnesium transporters, suggesting it is a new importer for magnesium. We demonstrate that this transport activity is likely to be specific rather than resulting from substrate promiscuity because the proteins are incapable of manganese import. This magnesium transport protein is distantly related to the Nramp family of proteins, which have been shown to transport divalent cations but have never been shown to recognize magnesium. We also find gene expression of the new magnesium transporter to be controlled by a magnesium-sensing riboswitch. Importantly, we find additional examples of riboswitch-regulated homologues, suggesting that they are a frequent occurrence in bacteria. Therefore, our aggregate data discover a new and perhaps broadly important path for magnesium import and highlight how identification of riboswitch RNAs can help shed light on new, and sometimes unexpected, functions of their downstream genes.

## Author Summary

Magnesium ions are essential for life, and, correspondingly, all organisms must encode for proteins to transport them. Three classes of bacterial proteins (CorA, MgtE and MgtA/B) have previously been identified for transport of the ion. This current study introduces a new route of magnesium import, which, moreover, is unexpectedly provided by proteins distantly related to Natural resistance-associated macrophage proteins (Nramp). Nramp metal transporters are widespread in the three domains of life; however, most are assumed to function as transporters of transition metals such as manganese or iron. None of the previously characterized Nramps have been shown to transport magnesium. In this study, we demonstrate that certain bacterial proteins, distantly related to Nramp homologues, exhibit transport of magnesium. We also find that these new magnesium transporters are genetically controlled by a magnesium-sensing regulatory element. Importantly, we find numerous additional examples of similar genes sharing this regulatory arrangement, suggesting that these genes may be a frequent occurrence in bacteria, and may represent a class of magnesium transporters. Therefore, our aggregate data discover a new and perhaps broadly important path of magnesium import in bacteria.

## Introduction

Metal ions are essential and serve many cellular purposes, including functioning as cofactors for numerous metalloenzymes. The latter are responsible for a diverse array of biochemical reactions and, together, comprise one third of all cellular proteins [1]–[3]. Conversely, all metals elicit toxic effects when they accrue to excess. Therefore, specific mechanisms are required for maintaining intracellular pools. In many instances, metal-sensing regulatory proteins (metalloregulatory proteins) control expression of transport proteins, or of metal-sequestering cellular factors [4]–[6].

One broadly important class of metal transporters is that of Nramps (natural resistance-associated macrophage proteins). The *Nramp1* family has been discovered in mammals to transport metals out of the macrophage phagosome. Mutational disruption of the gene results in increased susceptibility to infection by intracellular pathogens [7]–[9]. This suggests that deprivation of essential metals is a strategy used by hosts for compromising the phagosome as a niche for bacterial growth and replication. Nramp1 transports manganese, and possibly iron, while a second Nramp (Nramp2) transports primarily iron. In general, these Nramp genes are members of a large gene family, with numerous representatives in all three domains of life. For example, the sequence identity between bacterial and mammalian Nramps is high, often in excess of 35% [10]. Interestingly, just as mammalian Nramps are involved in microbial resistance, bacterial Nramps may be simultaneously required during infection by intracellular pathogens [11]–[13], although the importance of these proteins during infection remains a subject of debate [14], [15]. Essentially, bacterial and mammalian Nramps may compete for the same metals within the phagosome at the interface of host-pathogen interactions [16], [17]. Also, in addition to their important roles during microbial pathogenesis, genes encoding Nramps are required by many bacterial genomes as fundamental transporters of divalent ions.

Nramps share common structural features, including 10–12 transmembrane domains and conserved residues interspersed throughout. High-resolution structural data from X-ray crystallography is still lacking though, restricting knowledge of the structural basis of metal selectivity and transport. It is generally presumed that Nramp family members are employed for transport of manganese or iron, although some family members have been shown to exhibit broad transport activity for other divalent ions [18]–[21]. However, magnesium has not been shown to serve as a substrate for Nramps that have been characterized. Indeed, the initial characterization of a bacterial proton-dependent manganese transporter (MntH), which is a member of the Nramp family of proteins, demonstrated that it could import iron or manganese even in the presence of 5 mM magnesium, suggesting that magnesium was unlikely to serve as an MntH substrate [22].

Magnesium is the most abundant divalent metal in living cells and is required for numerous cellular activities, including serving as cofactor for enzymatic reactions and maintaining the structures of membranes and ribosomes [23]–[25]. Cytoplasmic levels of most transition metals are maintained at relatively low concentrations through action of high affinity metalloproteins [6], [26]–[28]. In contrast, intracellular free magnesium is maintained at a higher level (0.5–2.0 mM), which requires specific magnesium transport proteins [24], [29]–[33].

Three families of magnesium transporters have been discovered in bacteria: CorA, MgtE, and MgtA/MgtB P-type ATPase proteins [33]–[38]. While many metalloregulatory proteins have been identified as sensors of transition metals, less is known regarding control of magnesium homeostasis. One mechanism is through cytoplasmic gating domains of CorA and MgtE, which help couple intracellular magnesium demand with transport activity. In addition, a few genetic regulatory mechanisms have been discovered for magnesium homeostasis. Best studied in this regard is a two component regulatory system in *Salmonella enterica* that completes phosphoryl transfer from a sensor kinase (PhoQ) to response regulator (PhoP) in response to fluctuations in extracellular magnesium [39]. PhoP activates genes for magnesium homeostasis, such as *mgtA*, as well as genes important for growth and replication within a host cell. Interestingly, the *mgtA* transcript is also subject to a second, post-initiation layer of magnesium-responsive genetic regulation [40]. Changes in magnesium alter the secondary structure within the *mgtA* 5′ leader RNA; stabilization of one particular configuration is coupled with control of transcription elongation, which has the effect of limiting *mgtA* transcription to conditions of low magnesium [40].

Signal-responsive RNA elements, akin to *S. enterica mgtA*, which coordinate chemical cues with regulation of downstream gene expression, are referred to as riboswitches. A second, and mechanistically distinct, magnesium riboswitch, sometimes called the ‘M-box’, has also been discovered in bacteria. Originally discovered upstream of the *Bacillus subtilis mgtE* gene this riboswitch is also broadly conserved in numerous distantly-related bacteria [41]–[43]. It is almost always located upstream of one of the three known classes of magnesium transporters: CorA, MgtA, and MgtE. Riboswitches are generally composed of two portions: a signal-responsive aptamer and a downstream region that couples conformational changes of the aptamer with control of transcription, translation, or mRNA stability [44]–[48]. The structure of the magnesium-bound M-box aptamer domain has been resolved by X-ray crystallography and its mechanism for sensing magnesium has been investigated by various biochemical and biophysical experiments [49], [50]. Together, the aggregate data on this riboswitch suggest strongly that it serves as a metalloregulatory RNA for control of magnesium transport genes [42].

Given the close regulatory relationship between the M-box riboswitch and magnesium transporter genes, we were surprised to discover a subset of riboswitches situated upstream of *Nramp*-related genes. This observation established an intriguing conundrum. While M-box riboswitches respond to magnesium fluctuations in vivo, no Nramp or Nramp-related proteins have been found to transport this divalent ion. Therefore, one of these general assumptions must be incorrect. Either these particular riboswitches have been adapted to sense a divalent ion other than magnesium, or, alternatively, these particular Nramp-related homologs exhibit an unexpected role in magnesium homeostasis. Our combined data support the latter. We find that a *Clostridium acetobutylicum* ATCC 824 magnesium riboswitch controls expression of an Nramp-related gene and, moreover, that this particular transporter is surprisingly proficient in magnesium transport. These data, therefore, identify this subset of solute carrier proteins as a fourth class of magnesium transporters, designated herein as NrmT (Nramp-related magnesium transporter).

## Results

### Identification of magnesium riboswitches upstream of Nramp-related genes

**Figure 1:**
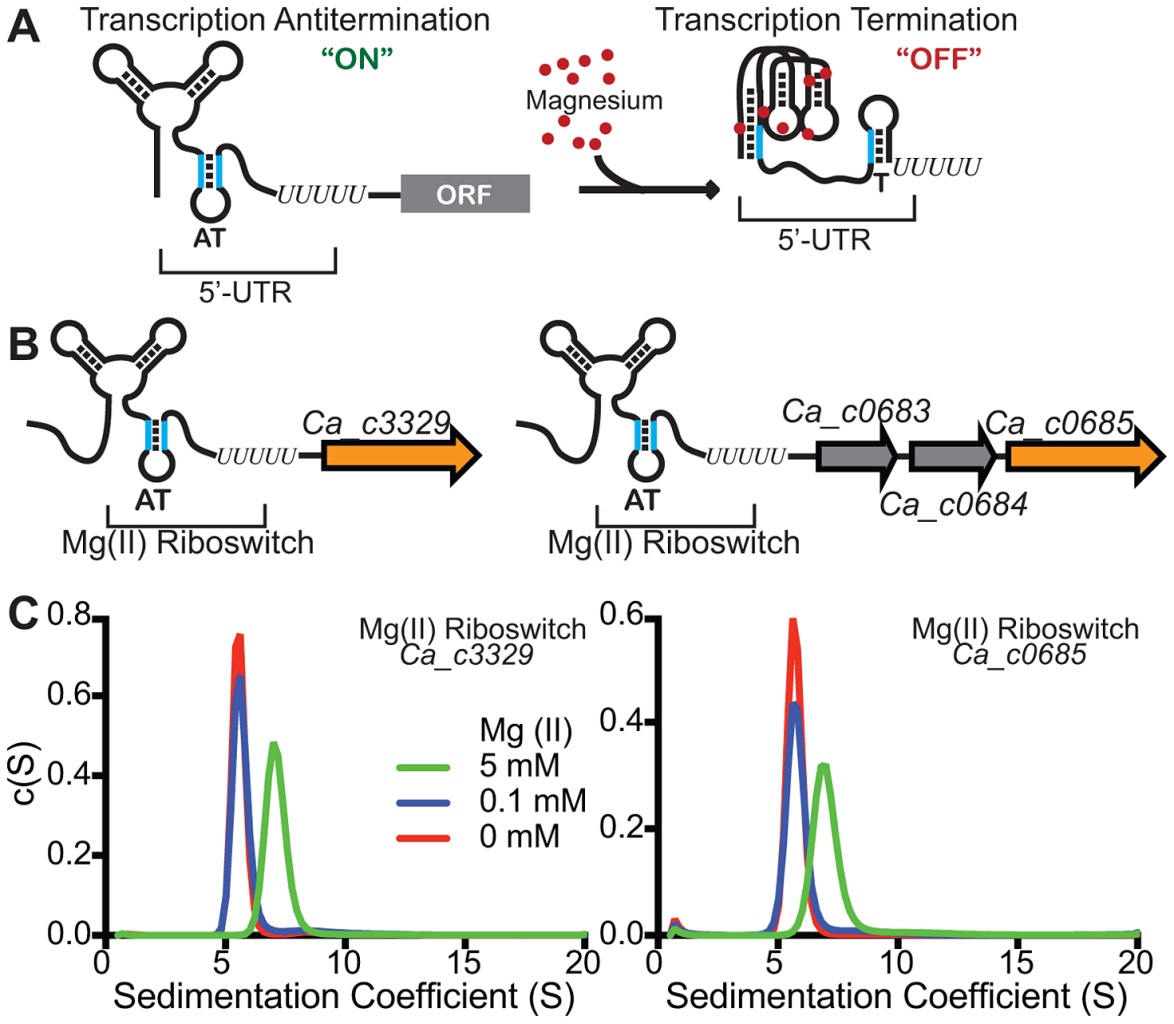
Identification of M-box RNAs located upstream of bacterial Nramp-related genes. (A) The magnesium riboswitch consists of two portions – a divalent-sensing aptamer and downstream sequences which couple the conformational status of the aptamer with formation of an intrinsic transcription termination site. An increase in intracellular magnesium triggers a compacted conformation of the aptamer domain [42] and sequestration of an oligonucleotide tract that would otherwise disrupt terminator formation (“T”). Therefore, increased magnesium promotes transcription termination, repressing downstream gene expression. (B) We searched for instances of putative magnesium riboswitches located upstream of Nramp-related genes and identified many such occurrences. Two were identified for *Clostridium acetobutylicum* Nramp-related sequences, *Ca_c0685* and *Ca_c3329*, and are shown schematically herein. (C) To determine whether the *Ca_c0685* and *Ca_c3329* RNA elements were likely to function as magnesium riboswitches, the respective aptamer domains were incubated with varying magnesium and analyzed by analytical ultracentrifugation. Prior studies of the magnesium riboswitch revealed a striking compaction of the aptamer domain in response to magnesium [41]–[42], [49]. The sedimentation velocity measurements of the *Ca_c0685* and *Ca_c3329* riboswitches revealed an identical compaction with magnesium, suggesting they are likely to function similar to the previously characterized riboswitch.

M-box magnesium riboswitches [41], [42] are widespread in bacteria, and are almost always positioned upstream of putative magnesium transport genes (*i.e.*, *corA*, *mgtE*, or *mgtA*). The riboswitch is presumed to control expression of the transport protein in a magnesium-responsive manner, as it does for *Bacillus subtilis mgtE* [41]. Most magnesium riboswitches affect gene expression by controlling formation of an intrinsic transcription terminator (Fig. 1A). A three-dimensional structural model of the aptamer (ligand-binding) domain revealed the presence of between 6 and 9 functionally important divalent ion binding sites [41], [49], [50]. However, this structural model alone cannot rule out the intriguing hypothesis that there might still exist aptamer variants that sense divalent ions other than magnesium. Motivated by this hypothesis, we searched using Infernal [51] for instances where M-box riboswitches were located upstream of genes for transport of metals other than magnesium. This search uncovered multiple instances where it appeared that putative Nramp family genes were located immediately downstream of M-box riboswitch candidates, mostly in Clostridia and Deltaproteobacteria. Since Nramp transporters are generally assumed to mediate transport of manganese and/or iron, and have never been show to transport magnesium, we chose to examine more closely a few representative examples of M box-regulated Nramp-related genes. For this, we chose two separate loci within the *Clostridium acetobutylicum* ATCC 824 genome (*Ca_c0685* and *Ca_c3329*) (Fig. 1B).

### Magnesium-specific regulation by the *Ca_c0685* riboswitch

**Figure 2:**
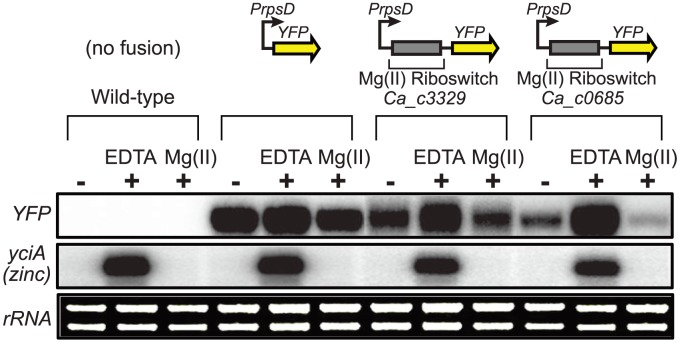
Control of gene expression by a *C. acetobutylicum* M-box RNA. The *Ca_c0685* and *Ca_c3329* riboswitches were fused downstream of a constitutive promoter (*PrpsD*) and upstream of the yellow fluorescent reporter gene (*yfp*) to determine whether they could control heterologous gene expression in a divalent cation-dependent manner. Control strains either lacking the *yfp* reporter construct or containing a constitutive *PrpsD-yfp* fusion were included in this study. Cells were cultured to mid-logarithmic growth phase in 2xYT rich medium supplemented with 50 µM MgCl_2_ (-), then incubated with 2 mM chelating agent, EDTA, or incubated in the presence of excess magnesium (2 mM) for one hour. Total RNA was assessed by staining of *rRNA* bands. Abundance of the *yfp* gene and of a zinc-responsive control transcript were monitored by S1 mapping. Radiolabeled DNA probes (Table S2) were used for S1 mapping of the *yfp* transcript.

**Figure 3:**
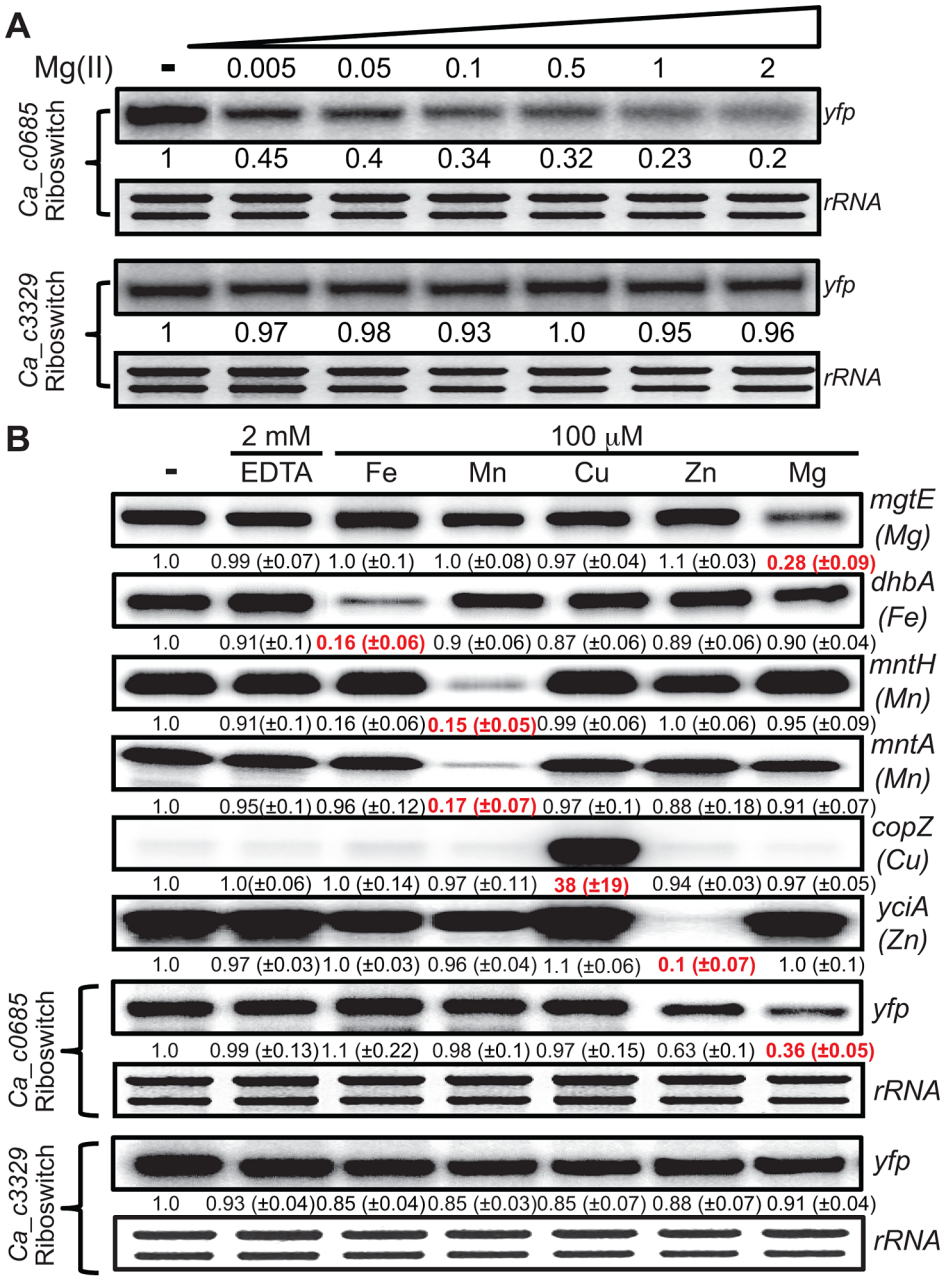
Metal specificity of *C. acetobutylicum* riboswitch-*yfp* reporter fusions. (A) The strains expressing either the *Ca_c0685* riboswitch*-yfp* or *Ca_c3329* riboswitch*-yfp* reporter fusions were cultured in glucose minimal medium supplemented with 50 µM magnesium until reaching an OD_600_ of ∼0.5–0.7, at which point 2 mM EDTA was added and cells were incubated for 1 hour. These cells were harvested by centrifugation and the pellet was washed three times and resuspended with an equal volume of chelated glucose minimum medium (chelated with Chelex-100). Either EDTA (2 mM final concentration) or varying magnesium concentrations were added and the cells were incubated for another 1 hour before harvesting. Total RNA was assessed by staining of *rRNA* bands. Abundance of the *yfp* gene was monitored by S1 mapping. (B) To assess the specificity of the *C. acetobutylicum* riboswitch-*yfp* reporter fusion, we cultured cells expressing either the *Ca_c0685 riboswitch-yfp* or *Ca_c3329 riboswitch-yfp* reporter fusions in glucose minimal medium supplemented with 50 µM magnesium and appropriate antibiotics until reaching an OD_600_ of ∼0.5–0.7, at which point 2 mM EDTA was added for 1 hour. These cells were harvested by centrifugation and the pellet was washed three times and resuspended with an equal volume of chelated glucose minimum medium (chelated with Chelex-100). Either EDTA (2 mM final concentration) or 100 µM various metals were added and the cells were incubated for another 1 hour before harvesting. Radiolabeled DNA probes (Table S2) were used for S1 mapping of the *yfp* transcript, and for several control transcripts that are known to respond to other metals (*e.g.*, *mgtE* (magnesium), *dhbA* (iron), *mntH* (manganese), *mntA* (manganese), *copZ* (copper), *yciA* (zinc)). These data demonstrate that the *Ca_c0685* specifically controls gene expression in response to magnesium, although addition of zinc also resulted in a moderate reduction in gene expression. Shown is a representative gel with quantification derived from experimental triplicates.

As a biochemical test of magnesium riboswitch function, the aptamer portions of the putative *Ca_c0685* and *Ca_c3329* riboswitches were transcribed in vitro and subjected to analytical ultracentrifugation measurements (Fig. 1C). Previous data using this technique demonstrated a large, and characteristic, change in hydrodynamic radius for *B. subtilis* magnesium riboswitches in response to binding of magnesium [41], [42], [49]. For example, the aptamer domain of the *B. subtilis* magnesium riboswitch exhibited a Svedberg coefficient of approximately 5.6 in low (100 µM) magnesium, and approximately 6.9 under conditions of elevated (5 mM) magnesium, indicating significant divalent ion-induced compaction. Sedimentation velocity measurements of the *Ca_c0685* and *Ca_c3329* riboswitch aptamer domains revealed strikingly similar changes in sedimentation coefficients upon addition of magnesium (*e.g.*, 5.8 and 7.0 in low and high magnesium, respectively, for Ca_c0685), consistent with significant metal-induced compaction of the Clostridial RNAs. Therefore, the *Ca_c0685* and *Ca_c3329* riboswitches appear by this biochemical test to resemble previously characterized magnesium riboswitches. For an in vivo test of riboswitch function, the *Ca_c0685* and *Ca_c3329* riboswitches were sub-cloned downstream of a constitutive promoter (*PrpsD*), but upstream of a yellow fluorescent reporter gene (*yfp*), for examination of their regulatory activity in vivo. The reporter fusions were integrated single copy into the *Bacillus subtilis* genome and *yfp* abundance was measured by S1 mapping (Fig. 2). Total RNA was extracted from cells cultured in rich media, after treatment for one hour by 2 mM EDTA or 2 mM magnesium chloride. A *PrpsD*-*yfp* control revealed almost no change in *yfp* upon treatment. In contrast, the *Ca_c0685* riboswitch-*yfp* fusion exhibited an increase in *yfp* upon EDTA treatment, and a reduction in *yfp* in response to 2 mM magnesium, consistent with regulatory control by a magnesium riboswitch. To explore this further, cells were cultured to mid-logarithmic growth phase and exposed to 2 mM EDTA, followed by resuspension in medium containing a range of magnesium concentrations (Fig. 3A). Under these conditions, the *yfp* transcript was increasingly reduced by the *Ca_c0685* riboswitch as extracellular magnesium was increased. However, only minor repression was observed with *Ca_c3329*.

In order to examine metal specificity in vivo for regulation by the *Ca_c0685* riboswitch, cells were cultured to mid-logarithmic growth phase, exposed to 2 mM EDTA, and resuspended in medium containing excess iron, manganese, copper, zinc or magnesium (Fig. 3B). Expression of the *Ca_c0685-yfp* reporter was evaluated alongside control measurements of *B. subtilis* metal transport genes. This analysis confirmed a three-fold reduction of *yfp* in response to magnesium by the *Ca_c0685* riboswitch. Excess zinc also moderately reduced the *yfp* transcript (∼37%); however, excessive levels of iron, manganese, or copper had no effect on *yfp*, although the presence of these metals did affect transcripts known to be under their regulatory influence. These data together revealed that the *Ca_c0685* riboswitch is a magnesium-sensing regulatory element. In contrast, it remains unclear from these experiments why the *Ca_c3329* riboswitch appears to be unresponsive within the *B. subtilis* host organism.

### Expression of *Ca_c0685* and *Ca_c3329* does not complement a deficiency in manganese transport

**Figure 4:**
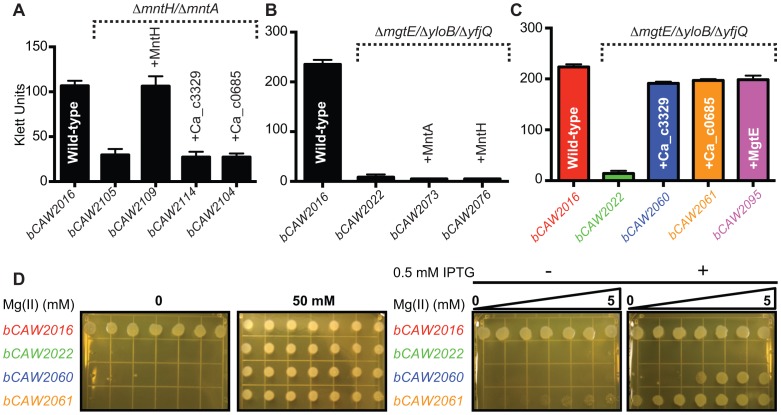
Heterologous expression of Ca_c0685 and Ca_c3329. The *ca_c0685* and *ca_c3329* genes were subcloned under IPTG-inducible control and integrated single-copy into the *B. subtilis amyE* gene. These expression cassettes were also integrated into various strains containing deletions of different divalent cation transporters. In all instances, expression of *ca_c0685* and *ca_c3329* was monitored by S1 mapping (Figure S1, S2, S3). To investigate the effect of gene expression in these various strains, cells were cultured alongside control strains. Shown herein are bar graphs plots of stationary phase growth for these respective strains. (A) Growth after entry into stationary phase is shown for *B. subtilis* control strains, including a wild-type and a manganese transport-deficient strain, and transport-deficient strains complemented with IPTG-inducible control of *B. subtilis* MntH, Ca_c0685 or Ca_c3329. These strains were cultured in minimal medium with no added manganese in the presence of 0.5 mM IPTG. (B) Heterologous expression of *B. subtilis* MntH and MntABCD do not rescue a magnesium-transport deficient phenotype. Growth measurements immediately after entry into stationary phase are shown for *B. subtilis* control strains, including wild-type, a magnesium transport-deficient strain, and transport-deficient strains complemented with either IPTG-inducible MntABCD or MntH. Full growth curves are shown in Figure S2. These strains were cultured in rich medium in the presence of 0.5 mM IPTG. (C) Heterologous expression of Ca_c0685 and Ca_c3329 in a magnesium transport-deficient strain. Growth measurements immediately after entry into stationary phase (full growth curves are included in Figure S3) are shown for *B. subtilis* strains, including wild-type, a magnesium transport-deficient control strain, and transport-deficient strains complemented with inducible Ca_c0685, Ca_c3329, or the magnesium transporter MgtE. The strains were cultured in rich medium in the presence of 0.5 mM IPTG and 2.5 mM magnesium. Expression of Ca_c0685 and Ca_c3329 both fully rescued growth in this medium. (D) In addition to the liquid culture growth experiments, 3 µl of each of these strains (∼1×10^4^/µl) was spotted onto solid medium containing a gradient of magnesium that ranged from 0 to 5 mM magnesium, respectively. These plates were incubated for 10 hours at 37°C before they were photographed.

Prior experimental evidence has primarily demonstrated that many bacterial Nramp homologues are for transport of manganese or iron [22], [52]–[56]. Although the Ca_c00685 and Ca_c3329 proteins are only distantly related to Nramp family proteins, the genes encoding Ca_c0685 and Ca_c3329 were heterologously expressed in *B. subtilis* under IPTG-inducible control (Fig. S1). To investigate a potential role in manganese transport, markerless deletion mutants of *B. subtilis* manganese transport genes, *mntH* and *mntABCD*, were introduced into these strains. The Δ*mntH/*Δ*mntABCD* double mutant (bCAW2105) exhibited a growth defect in defined medium, which could be rescued with addition of >10 µM manganese, or by ectopic expression of *B. subtilis* MntH (Fig. 4A; Fig. S1), consistent with prior findings [50]. In contrast, neither heterologous expression of Ca_c0685 or Ca_c3329 was able to rescue the manganese transport deficiency. Therefore, these *C. acetobutylicum* genes are unlikely to encode for manganese transport.

### Complementation of magnesium transport activity by *Ca_c0685 and Ca_c3329*

*B. subtilis* contains examples of all three magnesium transporter families [33]–[39], including *ykoK* (*mgtE* homolog), *yloB* (*mgtA* homolog), and two *corA* homologues (*yfjQ*, *yqxL*). A triple mutant (bCAW2022) containing markerless deletions of *mgtE*, *yloB*, and *yfjQ* exhibited a strong defect in magnesium transport activity (Fig. 4B; Fig. S2), and requires ∼50 mM extracellular magnesium to restore growth in rich medium [57]. As a preliminary check of the specificity of this magnesium transport-deficient phenotype, the known *B. subtilis* manganese transporters, *mntH* and *mntABCD*, were ectopically integrated into the genome under inducible control (creating bCAW2073 and bCAW2076). Expression of these manganese transporters was unable to complement the severe magnesium deficiency exhibited by bCAW2022. This supports the hypothesis that the bCAW2022 strain exhibits a specific defect in magnesium transport activity.

To examine the impact of Ca_c0685 and Ca_c3329 expression on magnesium transport, they were integrated single-copy into the bCAW2022 genome under inducible control and growth was assessed under conditions of magnesium limitation. Expression of Ca_c0685 fully restored growth to resemble that of wild-type cells (Fig. 4C; Fig. S3). Moreover, when this strain was inoculated onto solid medium that contained a gradient of magnesium from sub-micromolar to 5.0 mM, growth was observed on all portions of the plate, in contrast to bCAW2022 (Fig. 4D; Fig. S3). This suggests that Ca_c0685 rescued growth even under conditions of sub-micromolar magnesium. Also, Ca_c3329 was able to rescue the magnesium transport-deficient phenotype for bCAW2022; however, it rescued growth only when magnesium was included at >2 mM. Therefore, these data demonstrated that both Ca_c0685 and Ca_c3329 are capable of magnesium transport activity, with Ca_c0685 potentially showing higher affinity for the magnesium ion.

### Magnesium repression of *Ca_c0685 and Ca_c3329* in *Clostridium acetobutylicum*

**Figure 5:**
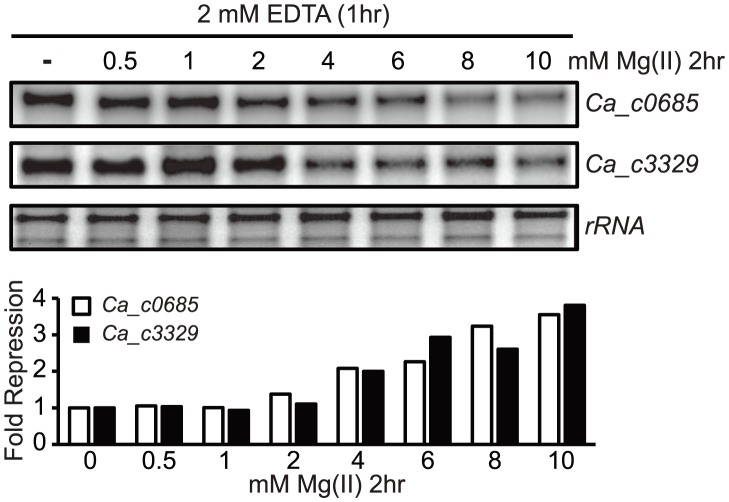
Magnesium repression of *Ca_c0685* and *Ca_c3329* in *C. acetobutylicum*. *C. acetobutylicum* cells were cultured in growth medium to an OD_600_ of ∼0.8. They were then treated with 2 mM EDTA for one hour, followed by centrifugation and resuspension in magnesium-free growth medium. The cells were then aliquoted into growth medium containing a range of magnesium concentrations and incubated for 2 hours under standard conditions. Abundance of the *Ca_c3329* and *Ca_c0685* transcripts was then measured by S1 mapping, as shown by the representative experiment herein.

Our observation that Ca_c3329 acts as a magnesium transporter seems at first glance to be inconsistent with our prior result showing that the *PrpsD*-*Ca_c3329*-*yfp* fusion was unresponsive to magnesium (or any other divalent ions tested). However, heterologous expression of riboswitch-reporter fusions is not always successful. This is, in certain instances, likely to be due to differences in the molecular environment, such as changes in RNase preferences or in RNA recognition by transcription elongation factors. Therefore, it is possible that the *Ca_c3329* riboswitch is nonfunctional when expressed in *B. subtilis* but is still functional in the *C. acetobutylicum* host for regulation of a transporter. However, it is also possible that the *Ca_c3329* riboswitch is nonfunctional in both organisms. As a test of these possibilities, *C. acetobutylicum* was cultured to OD_600_ of ∼0.8, and then treated with 2 mM EDTA for one hour, at which point the cells were harvested and resuspended in magnesium-free medium. These cells were then aliquoted into media containing varying magnesium concentration and incubated for 2 additional hours before extraction of total RNA. S1 mapping of *Ca_c0685* and *Ca_c3329* revealed that both genes were subjected to repression of transcription as magnesium was increased (Fig. 5). This indicates that both Ca_c0685 and Ca_c3329 are likely to be repressed by magnesium within the context of their host organism, and that the Ca_c3329 riboswitch is likely to be functionally responsive to magnesium in *C. acetobutylicum*.

### Phylogenetic analysis of Nramp-related transporters associated with magnesium riboswitches

**Figure 6:**
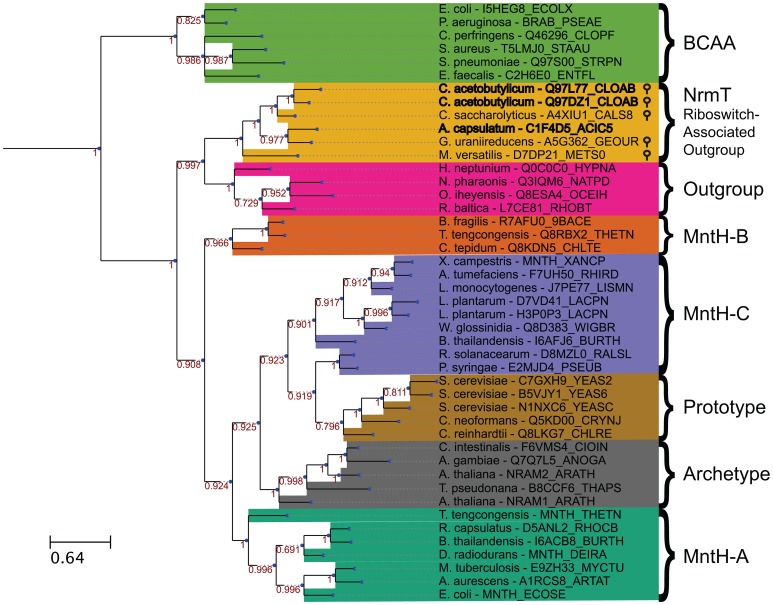
Bayesian phylogenetic tree of 45 Nramp family transporters, Nramp outgroup proteins, riboswitch-associated outgroup members, and branched-chain amino acid transporters. The sequences were aligned using MAFFT v7 using the L-INS-I algorithm [75]. This tree is the consensus of four replicate trees constructed using MrBayes 3.2 [76], [77]. Each replicate tree was constructed using four total chains for Metropolis coupling, and 1,000,000 generations with 25% relative burn-in. Priors used were the defaults for amino-acid models with an equal mixture of amino-acid substitution models. Transporter genes preceded by putative magnesium riboswitches are denoted by stem-loops. Branch support values are indicated in red by each internal node. Branch lengths represent expected number of substitutions per position. Similar trees were obtained with different approaches including maximum likelihood (MetaPIGA), minimum evolution, and maximum parsimony (MEGA6), not shown [78], [79].

Nramp family transporters are widespread among bacteria and eukaryotes [58] and are hypothesized to have emerged early in evolution. We performed a phylogenetic analysis of putative Nramp-related genes located immediately downstream of M-box RNAs. In addition, we collected representatives from three previously identified groups of bacterial MntH-like proteins (groups A, B and C, according to a previous classification, [58]), Nramp homologs from human, Arabidopsis and yeast, and several examples of an Nramp outgroup that was identified previously [59]–[61]. Members of the branched-chain amino acid transporter family, a part of the APC superfamily containing similar LeuT folds, were used as an outgroup, as in a previous characterization of Nramp phylogeny [60], [62]. We constructed the multiple sequence alignment (Fig. S4) and the maximum likelihood phylogenetic tree (Fig. 6) for the 47 selected representatives. All M-box-regulated homologs clustered into a single branch on the phylogenetic tree adjacent to the Nramp outgroup genes, whereas other bacterial Nramp transporters, including known manganese/iron transporters, were distributed in their respective groups. Interestingly, all Nramp-related transporters that appeared to be regulated by the magnesium riboswitch clustered together, suggesting a relationship between magnesium and members of this branch. This analysis indicates that these riboswitch-associated Nramp-related transporters form a distinct clade that is derived from a more distant common ancestor than those of the Nramp family, but that shares a more recent common ancestor with the Nramp outgroup. Given the phylogenetic relatedness of these proteins, we renamed them NrmT, for Nramp-related magnesium transporter.

To search for unique features of NrmT group, we examined the genomic context of representative genes (Fig. S5). Surprisingly, this revealed that the local genomic context of most group members includes a common, additional gene. This latter gene appears by sequence homology to encode for a protein that is specifically homologous to the N-terminal cytoplasmic domain of the MgtE transporter. This protein appears to be encoded by a single gene, except in *C. acetobutylicum* where the riboswitch-regulated operon *Ca_c0685* includes a homolog that is split into two smaller genes (Fig. 1B).

Together, these observations appear to suggest a possible relationship between magnesium homeostasis and the Nramp-like outgroup (NrmT) identified herein. As a preliminary test of this possibility, another member of this grouping, but that lacked a magnesium riboswitch, was arbitrarily chosen for heterologous expression in *B. subtilis*. Specifically, we identified an *nrmT* gene from *Acidobacterium capsulatum*, located downstream of the small gene exhibiting homology to the MgtE cytoplasmic domain. Heterologous expression of the *A. capsulatum* Nramp-related gene (Fig. S6) in bCAW2022 (*i.e.*, the magnesium transporter-deficient strain) rescued growth, but only in the presence of low millimolar magnesium. The partial MgtE gene was then integrated at a separate locus of the genome and co-expressed with the *A. capsulatum* putative transporter; expression of both proteins did not further improve rescue of the magnesium transport defect. However, expression of this *A. capsulatum* transporter gene was fully capable of rescuing the manganese transport defect exhibited by bCAW2105 (Δ*mntH/*Δ*mntABCD*) (Fig. S6). These data suggest that the *A. capsulatum* NrmT protein is likely to function as a manganese transporter, although what role the partial MgtE gene may play in metal transport remains unknown. Therefore, only a subset of the NrmT proteins, sometimes associated with magnesium riboswitches, is likely to exhibit high affinity magnesium transport. These data also illustrate the potential value of using the magnesium riboswitch as an identifying feature of magnesium specificity in associated transporters.

## Discussion

The three major classes of bacterial magnesium transporters (CorA, MgtE, MgtA) were discovered using complementation strategies similar to that described herein [35], [63]–[65]. While other, minor routes of magnesium import may be possible, organisms from all three domains of life are generally expected to encode at least a subset of these three protein families, as magnesium acquisition is essential. In this study, we employed a similar complementation approach to suggest that *C. acetobutylicum* proteins, unrelated to the known classes of transporters, are capable of magnesium transport. As these proteins are distantly related to the Nramp family of proteins, which have not been found to transport magnesium, this was an unexpected discovery that suggests either the substrate range for Nramp transporters must be expanded to include this divalent ion, or, more likely, that a new class of Nramp-related divalent metal transporters has been introduced. Therefore, these observations together suggest that a subset of Nramp-related transport proteins constitutes a fourth class of dedicated magnesium transporters in bacteria, designated herein as NrmT.

Our data also revealed that a riboswitch upstream of *Ca_c0685* is proficient within the confines of a heterologous host in coupling intracellular magnesium fluctuation with control of downstream gene expression. This observation strengthens the overall body of evidence showing that magnesium is the central signal perceived by the M-box riboswitch. It also suggests that identification of these riboswitches can, in certain instances, be used to help predict which Nramp-related homologues are likely to function as dedicated magnesium transporters, rather than transporters of other divalent cations such as manganese. This is also bolstered by our observations that *Ca_c3329* was both repressed by magnesium in *C. acetobutylicum* and provided magnesium transport activity in *B. subtilis*, albeit at higher concentrations of the ion. Therefore, in total, we speculate that the *C. acetobutylicum* magnesium riboswitches control expression of two magnesium-transporting Nramp-related genes, which may be functionally specialized for different ranges of extracellular magnesium. There are two MntH homologues that are also encoded by this organism (*Ca_p0063* and *Ca_c0628*, representing a MntH-A and MntH-B protein, respectively) [55], which are not associated with magnesium riboswitches and that we speculate are likely to provide transport of manganese or iron.

MntH homologues are widespread members of the Nramp family of proteins. In addition to two groups of eukaryotic Nramps, phylogenetic analyses have identified three groups of bacterial MntH proteins, designated as group A, B, and C. The group A proteins were characterized as proton motive force-dependent transporters of divalent ions, typically manganese or iron [20], [22], [53], [59], [66]. Also, group A MntH genes have been shown to be expressed by intracellular bacteria during host infection, and to be regulated by external availability of metal ions by the manganese-responsive MntR repressors [22], [53]. Group C MntH share a closer sequence relationship with eukaryotic Nramp homologues, while group B exhibits an origin closer to the root of the Nramp tree. Group B MntH derive mostly from strict anaerobes, such as *Chlorobium*, proto-photosynthetic bacteria and *Clostridium* species, suggesting it may have appeared before onset of aerobiosis [55], [58]. In contrast, the *C. acetobutylicum* transporters characterized herein do not belong in any of these Nramp subgroups (Fig. 6). Instead, they display only a moderate relationship to previously established Nramp groups. Their closest relationship to established Nramp groupings is with group B members; however, there is still considerable distance exhibited between them. Instead, phylogenetic analysis indicates that the magnesium riboswitch-regulated proteins are more closely related to a phylogenetic outgroup of the Nramp family than to the family itself. This outgroup was previously identified [61] as an evolutionary intermediate between the Nramp family and a superfamily characterized by the LeuT 3D fold [62]. The taxonomic distribution of this particular outgroup is broad and is not restricted to species where magnesium riboswitches are associated. When analyzed with the phylogenetic data, a structural modeling analysis previously suggested that the Nramp phylogenetic outgroup segregates between the large cluster of transporters known to share the LeuT 3D fold and the Nramp family [61], [67]. Based on this work [61], [67] the Nramp outgroup may be viewed as an evolutionary link between cation-driven transporters that act on non-metal substrates and those transporting divalent metals. In this study, we find that the riboswitch-regulated proteins are related to this previously described outgroup, but may also form their own subclass (Fig. 6).

It is not yet obvious what sequence features might differentiate the magnesium-transporting proteins from Nramp-related proteins transporting other divalent ions. However, prior multiple sequence alignments suggested a few amino acids that are conserved and that may be involved in metal coordination. For example, several individual intramembrane sites that distinguish Nramps from the outgroup have been implicated in cation transport [61]. Some of these residues are maintained while others are replaced in the proteins associated with magnesium riboswitches (Fig. S4). Therefore, subsequent site-directed analyses of these and other residues may eventually provide important insight into the metal selectivity and transport cycling properties exhibited by this newly discovered magnesium-transporter.

Further study is also required for determining the functional role(s) of the ancillary gene, which encodes for a small protein homologous to the N-terminal cytoplasmic domain of the MgtE transporter. This ORF was not strictly required for magnesium transport activity of Ca_c0685 and Ca_c3329, as it was not included in our genetic complementation assays; however, it may play an additional role. The cytosolic domain of MgtE has been suggested by prior biochemical and structural data to contain magnesium sites that regulate transport activity. From this, we speculate that the MgtE-like fragment is likely to provide cytoplasmic feedback regulation of transport for the NrmT proteins, or, alternatively, may affect metal chaperone activity. A role for the MgtE-like fragment in metal homeostasis is also supported by the observation that many of the MgtE-like fragments are regulated by magnesium riboswitches, co-transcribed with the NrmT outgroup members. However, our complementation analysis of the *A. capsulatum* outgroup protein, which lacks a magnesium riboswitch, revealed that it is likely to primarily transport manganese, rather than magnesium, despite the presence of an adjacent MgtE-like fragment. Therefore, the MgtE-like fragment that is associated with the *A. capsulatum* protein would be expected to serve a function other than magnesium regulation, perhaps instead responding to manganese ions. Subsequent biochemical analysis of transport activity will be required to test these predictions, and to reveal the function of the small ORF.

Nramps are believed to be important in most organisms for transport of manganese or iron. We demonstrate herein that an outgroup of Nramp-related proteins are likely to function as dedicated magnesium transporters. Therefore, when considering the potential routes of magnesium transport activity for a target organism, this family of proteins must be considered as potential suspects, along with previously identified magnesium transport classes. Future studies will be required to compare these newly discovered magnesium transporters with CorA, MgtE and MgtA/B proteins, and to determine whether they are also important to infection by bacterial pathogens.

## Materials and Methods

### Strains and culture conditions

All *B. subtilis* strains used in this study were isogenic with common laboratory strains listed in Table S1. Depending on the experiment, they were cultured in liquid rich medium [2xYT; (16 g/L tryptone, 10 g/L yeast extract, 5 g/L NaCl)], solid rich medium [Tryptone Blood Agar Base (TBAB)], and glucose minimal medium [20 g/L (NH_4_)_2_SO_4_, 183 g/L K_2_HPO_4_*3H_2_O, 60 g/L KH_2_PO_4_, 10 g/L sodium citrate, 0.5% glucose, 0.5 mM CaCl_2_, 5 µM MnCl_2_, and 2 g/L MgSO_4_*7H_2_O when appropriate] at 37°C. When appropriate, antibiotics were included at: 100 µg/mL spectinomycin, 5 µg/mL chloramphenicol and 1 µg/mL erythromycin plus 25 µg/mL lincomycin. To chelate divalent cations from media, 5 g/100 mL of Chelex-100 resin (Bio-Rad) was added to dissolved medium and equilibrated with stirring for 2 hours, followed by removal of resin by filtration. DNA was transformed into *B. subtilis* using a modified version of a previously published protocol [68].

### Construction of riboswitch-*yfp* reporter strains

For construction of reporter fusions between *C. acetobutylicum* riboswitches and yellow fluorescent protein gene (*yfp*), the putative magnesium riboswitches were amplified by PCR and subcloned into pDG1662 (Table S2). The constitutive promoter from *B. subtilis rpsD* was subcloned upstream while the *yfp* gene was placed downstream of the putative riboswitches, respectively. These plasmids were transformed into *B. subtilis* PY79 for integration into *amyE*.

### Construction of strains for complementation experiments

The wild-type strain for all complementation experiments was derived from *B. subtilis* 168 by integration of empty pHyperspank vector at the *amyE* locus [69]. Manganese and magnesium transporter knockout strains were created by in-frame markerless deletion [57], [70]. Complementation strains were created by sub-cloning of the transporter genes into pHyperspank under the control of an IPTG-inducible promoter and double homologous recombination of the resulting construct into the *amyE* locus of the appropriate transporter knockout strain. Correct strains were verified by diagnostic PCR and Sanger sequencing. All strains used in this experiment are listed in Table S1.

### Growth measurements

*B. subtilis* strains were cultured on TBAB plates, supplemented when necessary with MgCl_2_ concentrations indicated in the text, and the appropriate antibiotics. These cells were used to inoculate 5 mL of either 2xYT or glucose minimal media (GMM) [43], that was cultured overnight while shaking at 37°C. An aliquot was then diluted 1∶100 in 25 mL 2xYT or GMM supplemented as necessary with antibiotics. These cells were incubated shaking at 37°C until reaching an OD_600_ of ∼0.5, whereupon they were pelleted, washed twice with 10 mL 2xYT or GMM, and resuspended to an OD_600_ of ∼0.05 in 25 mL, including the indicated amounts of MgCl_2_ and/or 0.5 mM IPTG. Klett readings were recorded at regular time intervals using 250 mL flasks. Stationary phase OD_600_ measurements were taken at the second time point after the intersection of exponential and stationary growth phases.

### Gradient plate assays

For preparation of agar plates containing a gradient of magnesium, we utilized a procedure described previously [71], [72]. Briefly, a slanted 2% agar medium base containing the maximum desired concentration of magnesium was prepared in a standard petri dish, upon which a magnesium-free, 0.8% top agar medium was poured, thus allowing a gradient of magnesium to be established by diffusion from the slanted bottom layer. Approximately 3 µl culture (at ∼1×10^4^ cells/µl) were spotted onto magnesium gradient plates (from 0 mM magnesium to either 2.5 or 5.0 mM magnesium), with or without 0.5 mM IPTG and incubated for exactly 10 hours at 37°C at which point the plates were imaged by photography. In a related experiment, a serially diluted culture (from 6.25×10^3^ to 100 cells) was spotted onto glucose minimal medium plates with and without 10 µM manganese chloride, and with and without 0.5 IPTG, and incubated for exactly 10 hours at 37°C at which point they were photographed.

### S1 mapping analysis

Total RNA was harvested from cells that were grown to mid-logarithmic phase in 2xYT or in glucose minimal medium [41]. When appropriate, the glucose minimal medium was first subjected to chelation of divalent metals by incubation with various amounts of EDTA, as described in the manuscript. Total RNA was extracted by hot phenol after fixation of cell pellet with RNAprotect reagent (Qiagen), according to the manufacturer instructions and as described previously [28], [73]. The quality and quantity of RNA was measured by absorbance spectroscopy and confirmed by resolution on 1.3% formaldehyde-agarose gels. Gene-specific oligonucleotide probes (Table S2) for *Ca_c3329*, *Ca_c0685*, *mntA*, *mntH*, *yfp*, *mgtE*, *dhbA*, *copZ*, and *yciA* transcripts were used for PCR amplification using *Clostridium acetobutylicum* and *B. subtilis* genomic DNA as template. Each specific DNA probe was radiolabeled with [γ-^32^P] ATP and T4 polynucleotide kinase and 30,000–40,000 cpm of labeled probe was used in each reaction. 100 µg of total RNA was pelleted and lyophilized; this pellet was then carefully resuspended in 20 µl hybridization buffer [40 mM PIPES (pH 6.4), 400 mM NaCl, 1 mM EDTA, 80% (v/v) formamide]. Individual samples were incubated at 80°C for 25 min and slow cooled to 42°C. 300 µl of S1 nuclease mix (∼100 units in S1 nuclease buffer [280 mM NaCl, 30 mM NaOAc (pH 4.4), 4.5 mM ZnOAc]) was added and incubated at 37°C for 45 min. The reaction was terminated by addition of 75 µl of S1 nuclease termination solution (2.5 M NH_4_OAc, 0.05 M EDTA). The DNA-RNA hybrid was precipitated by adding 400 µl of isopropanolol and the pellet was washed with 70% (v/v) ethanol, vacuum dried, and resuspended in 10 µl alkaline loading dye. The protected DNA fragments were then resolved by 6% (wt/vol) polyacrylamide gels containing 7 M urea. The dried gels were exposed to a phosphor imaging screen (FLA-2000; Fuji) and bands were quantified using Multi Gauge V3.0 or ImageJ.

### Growth of *Clostridium acetobutylicum*

*C. acetobutylicum 824* was cultured in 400 mL Clostridial growth medium (CGM) [74] until it reached an OD_600_ of ∼0.8. EDTA (pH 8.0) was added to a final concentration of 2 mM. After one-hour incubation, the cells were harvested by centrifugation and resuspended in magnesium-free CGM. 40 mL of this cell suspension was then aliquoted into separate containers containing designated concentrations of magnesium. After a two-hour incubation, cells were harvested by centrifugation and stored at −80°C until lysis and RNA extraction.

## Supporting Information

Figure S1 Expression of *Ca_c0685* and *Ca_c3329* in a manganese deficient strain. (A) Genotype of strains (Table S1). (B) Expression of *Ca_c0685* and *Ca_c3329* in a manganese-deficient strain. Strains containing inducible control of Ca_c0685 and Ca_c3329 were created as described in the text and analyzed alongside control strains. 0.5 mM IPTG was added to exponentially growing cultures for 1 hr, whereupon 100 µg of total RNA was hybridized with the appropriate radiolabeled S1 probe DNA. DNA oligonucleotides used for S1 mapping are listed in Table S2. “+” indicates addition of IPTG, whereas “−” indicates the absence of IPTG. Following S1 mapping, the protected DNA probes were analyzed by phosphor imaging. Representative results are presented in this figure. These data indicate that the *Ca_c0685* and *Ca_c3329* genes are transcribed under these conditions. (C) Growth curves are shown for *B. subtilis* control strains, including wild-type and a Δ*mntH/*Δ*mntABCD* manganese-deficient double mutant, and transport-deficient strains containing IPTG-inducible copy of *mntH*, *Ca_c0685*, or *Ca_c3329* integrated into the *amyE* locus. These strains were cultured in minimal medium without added manganese in the presence of 0.5 mM IPTG. (D) They were also serially diluted onto solid growth medium that either contained or lacked 10 µM manganese, and that either contained or lacked 0.5 mM IPTG for induction of Nramp-related genes. Only bCAW2109, containing ectopic expression of MntH, was capable of rescuing growth on the manganese-limiting medium.
(PNG)

Figure S2 Heterologous expression of *B. subtilis* MntH and MntABCD do not rescue a magnesium-deficient phenotype. (A) Genotype legend (Table S1). (B) Expression of manganese transport genes, *mntH*, and *mntA*, within the context of a magnesium deficient strain. The transcripts for *mntA*, and *mntH* were examined by S1 mapping analysis for the strains mentioned in this figure and described in the text. Total RNA was extracted from exponentially growing cells after one hour of treatment with 0.5 mM IPTG (“+”) or in the absence of IPTG (“−”). Ethidium bromide-stained rRNA is included as a loading control in these analyses. DNA oligonucleotides used for S1 mapping are listed in Table S2. Following S1 mapping, the protected DNA probes were analyzed by phosphor imaging. Representative results are presented in this figure. These data indicate that the *mntH* and *mntA* genes are transcribed under these conditions. (C) Growth curves are shown for *B. subtilis* control strains, including wild-type and a Δ*mgtE/*Δ*yloB/*Δ*yfjQ* triple mutant that is deficient in magnesium transport activity, and transport-deficient strains containing an IPTG-inducible copy of the *mntH* or *mntABCD* genes. These strains were cultured in rich medium in the presence of 0.5 mM IPTG. (D) They were also serially diluted onto solid growth medium that either contained or lacked 50 mM magnesium, and that either contained or lacked 0.5 mM IPTG for induction of either MntH or MntABCD. The petri plates were incubated for 32 hrs at 37°C, at which point they were photographed. Only the wild-type strain grew in the absence of 50 mM magnesium. As further evidence, 3 µL of these strains (∼1×10^4^/µL) were spotted onto rich medium plates containing a gradient of magnesium ranging from 0 to 5 mM. Again, only wild-type grew under these conditions.
(PNG)

Figure S3 Heterologous expression of Ca_c0685 and Ca_c3329 in a magnesium transport-deficient strain. (A) Genotype legend (Table S1). (B) Strains containing inducible Ca_c0685 and Ca_c3329 analyzed alongside control strains. 0.5 mM IPTG was added to exponentially growing cultures for 1 hr, whereupon 100 µg of total RNA was hybridized with radiolabeled S1 probe DNA respectively. DNA oligonucleotides used for S1 mapping are listed in Table S2. “+” indicates addition of IPTG, whereas “−” indicates the absence of IPTG. Following S1 mapping, the protected DNA probes were analyzed by phosphor imaging. Representative results are presented in this figure. These data indicate that the *Ca_c0685* and *Ca_c3329* genes are transcribed under these conditions. (C) Growth curves are shown for *B. subtilis* control strains, including wild-type and a Δ*mgtE/*Δ*yloB/*Δ*yfjQ* triple mutant that is deficient in magnesium transport activity, and transport-deficient strains containing an IPTG-inducible copy of *Ca_c0685* or *Ca_c3329* integrated into the *amyE* locus. The resulting strains were cultured in rich medium in the presence of 0.5 mM IPTG and 2.5 mM magnesium. Expression of Ca_c0685 and Ca_c3329 both fully rescued growth in this medium. (D) Also, 3 µL of each of these strains (∼1×10^4^/µL) was spotted onto solid medium containing a gradient of magnesium that ranged from 0 to 2.5 mM magnesium. These plates were incubated for 10 hours at 37°C before they were photographed. These results revealed that Ca_c0685 fully rescued growth of the magnesium-deficient strain whereas Ca_c3329 only rescued growth in the presence of low millimolar magnesium.
(JPG)

Figure S4 Multiple sequence alignment of Nramp transporters. Magnesium-associated branch regulated by M-box riboswitches is in red font. Selected conserved residues that differ in the magnesium-associated genes or are important for manganese/iron uptake in MntH from *E. coli* [61] are indicated by annotation.
(PDF)

Figure S5 Genome context analysis of MgtE-associated branch of NrmT genes. M-box riboswitches were identified in the promoter regions of *mgtE-nramp* operons using the Rfam database of RNA motifs. Phylogenetic tree for the group of related proteins using was constructed using the MicrobesOnline genomic database. Experimentally tested transporters from *C. acetobutylicum* and *A. capsulatum* and are in red and blue, respectively.
(JPG)

Figure S6 Expression of *A. capsulatum* ACP2976 and ACP2977. (A) Schematic representation of the gene arrangement of a Nramp-related gene from *Acidobacterium capsulatum*. This particular Nramp relative (Acp2977) was chosen as it is related to the magnesium-transporting Ca_c0685 gene but lacks an observable magnesium riboswitch. However, it, like the majority of magnesium associated Nramp homologues is located immediately downstream of an open reading frame that appears to encode for a protein that is homologous to the cytoplasmic domain of the magnesium transporter, MgtE (Acp2976). (B) The *Acp2977* gene was integrated into the *B. subtilis amyE* gene while the *Acp2976* gene was integrated into the *sacA* locus under IPTG- and xylose-inducible control, respectively. The background *B. subtilis* strain also included deletions of three putative magnesium transporters, *mgtE*, *yloB*, and *yfjQ*. (C) Analysis of this and other control strains by S1 mapping showed that the *Acp2976* and *Acp2977* genes were indeed transcribed when induced by xylose and IPTG, respectively. (D) 3 µl of these strains (∼1×10^4^/µL) was spotted onto solid medium containing a gradient of magnesium from 0 to 5 mM magnesium. These plates were incubated for 10 hours at 37°C before they were photographed. These results revealed that the Nramp homologue, Acp2977, could partially rescue growth of the magnesium-deficient strain whereas Acp2976 alone was unable to rescue growth under these conditions. (E) Strains that included IPTG-inducible copies of either *Acp2977* or *Acp2976* and that included deletion of manganese transport genes were serially diluted onto solid growth medium that either contained or lacked 10 µM manganese. Several control strains are included in this analysis and are described in the figure. Under these conditions induction of *B. subtilis* MntH was sufficient for rescue of growth in the absence of added manganese. Similarly, induction of Acp2977 fully rescued growth in the absence of added manganese; therefore, the Acp2977 Nramp homologue is likely to function as a manganese transport protein.
(JPG)

Table S1 *Bacillus subtilis* strains and plasmids used in this study. Shown in this table are all of the strains and relevant plasmids for the experimentation described herein. The construction and characterization of the magnesium transporter deletion strains, including the marker-less *ΔmgtE*, *ΔyloB*, and *ΔyfjQ* deletion strains, are described in detail in a separate publication [57]. Similarly, construction and characterization of markerless deletions of *mntABCD*, and *mntH* are also described in this publication [57].
(DOCX)

Table S2 The DNA oligonucleotides that were used in this study are listed and briefly described herein.
(DOCX)
