# Supplementary figures and images for: Transport of Magnesium by a Bacterial Nramp-Related Gene
Source: PLoS Genet. 2014 Jun 26;10(6):e1004429. doi: 10.1371/journal.pgen.1004429 (PMC4072509; doi:10.1371/journal.pgen.1004429)

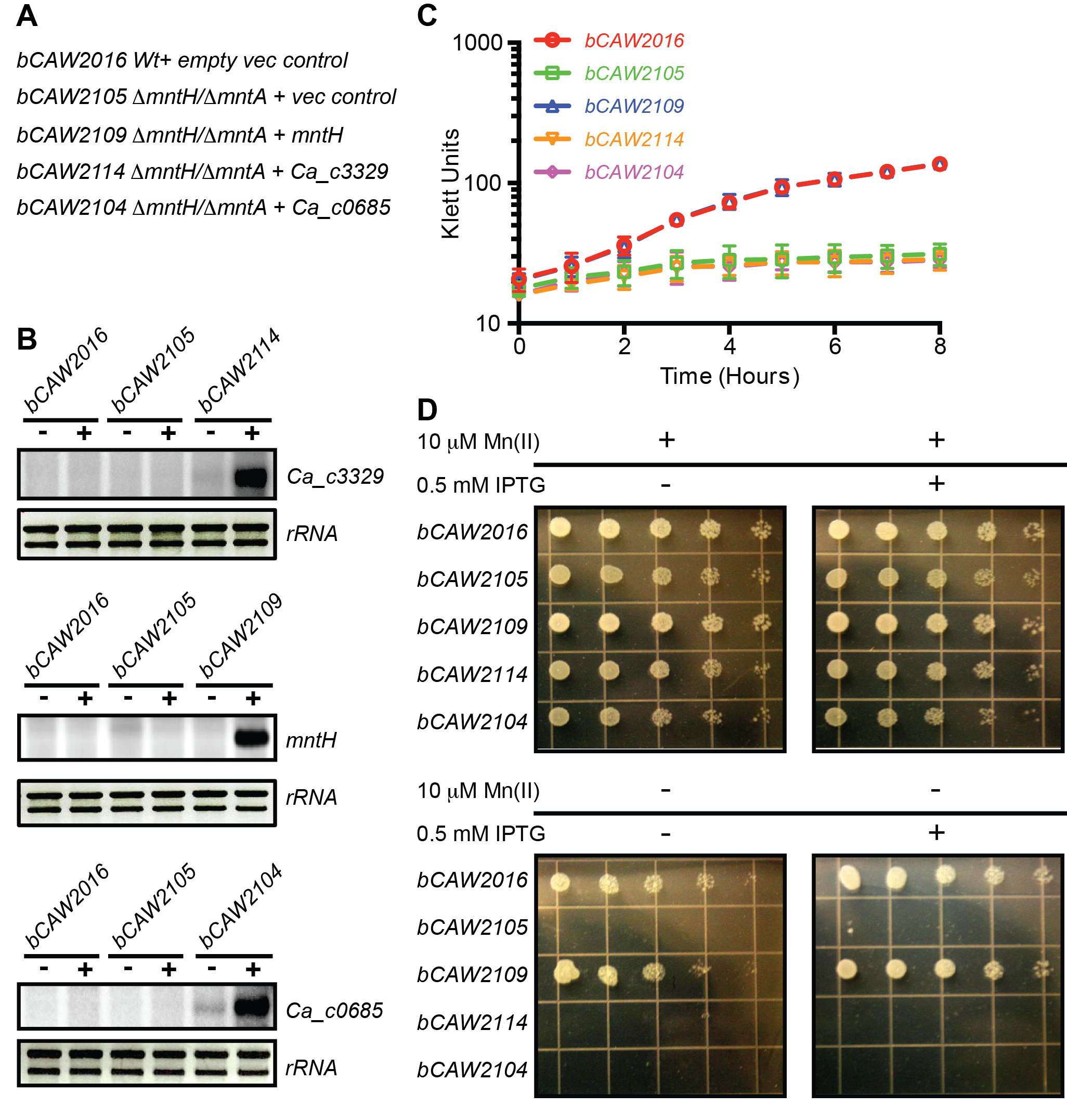

Supplement: Figure S1 — Expression of Ca_c0685 and Ca_c3329 in a manganese deficient strain. (A) Genotype of strains (Table S1). (B) Expression of Ca_c0685 and Ca_c3329 in a manganese-deficient strain. Strains containing inducible control of Ca_c0685 and Ca_c3329 were created as described in the text and analyzed alongside control strains. 0.5 mM IPTG was added to exponentially growing cultures for 1 hr, whereupon 100 µg of total RNA was hybridized with the appropriate radiolabeled S1 probe DNA. DNA oligonucleotides used for S1 mapping are listed in Table S2. “+” indicates addition of IPTG, whereas “−” indicates the absence of IPTG. Following S1 mapping, the protected DNA probes were analyzed by phosphor imaging. Representative results are presented in this figure. These data indicate that the Ca_c0685 and Ca_c3329 genes are transcribed under these conditions. (C) Growth curves are shown for B. subtilis control strains, including wild-type and a ΔmntH/ΔmntABCD manganese-deficient double mutant, and transport-deficient strains containing IPTG-inducible copy of mntH, Ca_c0685, or Ca_c3329 integrated into the amyE locus. These strains were cultured in minimal medium without added manganese in the presence of 0.5 mM IPTG. (D) They were also serially diluted onto solid growth medium that either contained or lacked 10 µM manganese, and that either contained or lacked 0.5 mM IPTG for induction of Nramp-related genes. Only bCAW2109, containing ectopic expression of MntH, was capable of rescuing growth on the manganese-limiting medium. (PNG) [file pgen.1004429.s001.png]

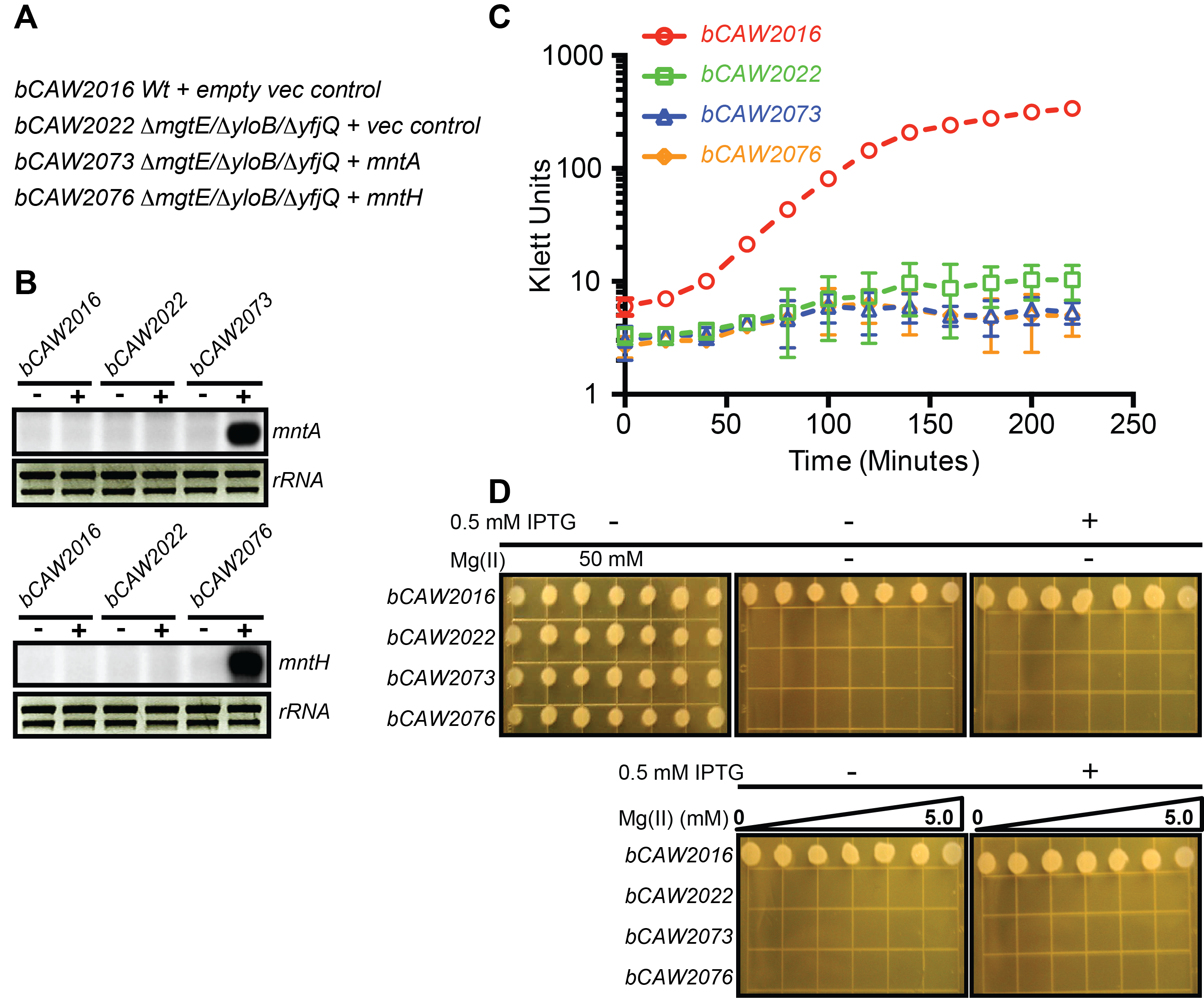

Supplement: Figure S2 — Heterologous expression of B. subtilis MntH and MntABCD do not rescue a magnesium-deficient phenotype. (A) Genotype legend (Table S1). (B) Expression of manganese transport genes, mntH, and mntA, within the context of a magnesium deficient strain. The transcripts for mntA, and mntH were examined by S1 mapping analysis for the strains mentioned in this figure and described in the text. Total RNA was extracted from exponentially growing cells after one hour of treatment with 0.5 mM IPTG (“+”) or in the absence of IPTG (“−”). Ethidium bromide-stained rRNA is included as a loading control in these analyses. DNA oligonucleotides used for S1 mapping are listed in Table S2. Following S1 mapping, the protected DNA probes were analyzed by phosphor imaging. Representative results are presented in this figure. These data indicate that the mntH and mntA genes are transcribed under these conditions. (C) Growth curves are shown for B. subtilis control strains, including wild-type and a ΔmgtE/ΔyloB/ΔyfjQ triple mutant that is deficient in magnesium transport activity, and transport-deficient strains containing an IPTG-inducible copy of the mntH or mntABCD genes. These strains were cultured in rich medium in the presence of 0.5 mM IPTG. (D) They were also serially diluted onto solid growth medium that either contained or lacked 50 mM magnesium, and that either contained or lacked 0.5 mM IPTG for induction of either MntH or MntABCD. The petri plates were incubated for 32 hrs at 37°C, at which point they were photographed. Only the wild-type strain grew in the absence of 50 mM magnesium. As further evidence, 3 µL of these strains (∼1×10^4^/µL) were spotted onto rich medium plates containing a gradient of magnesium ranging from 0 to 5 mM. Again, only wild-type grew under these conditions. (PNG) [file pgen.1004429.s002.png]

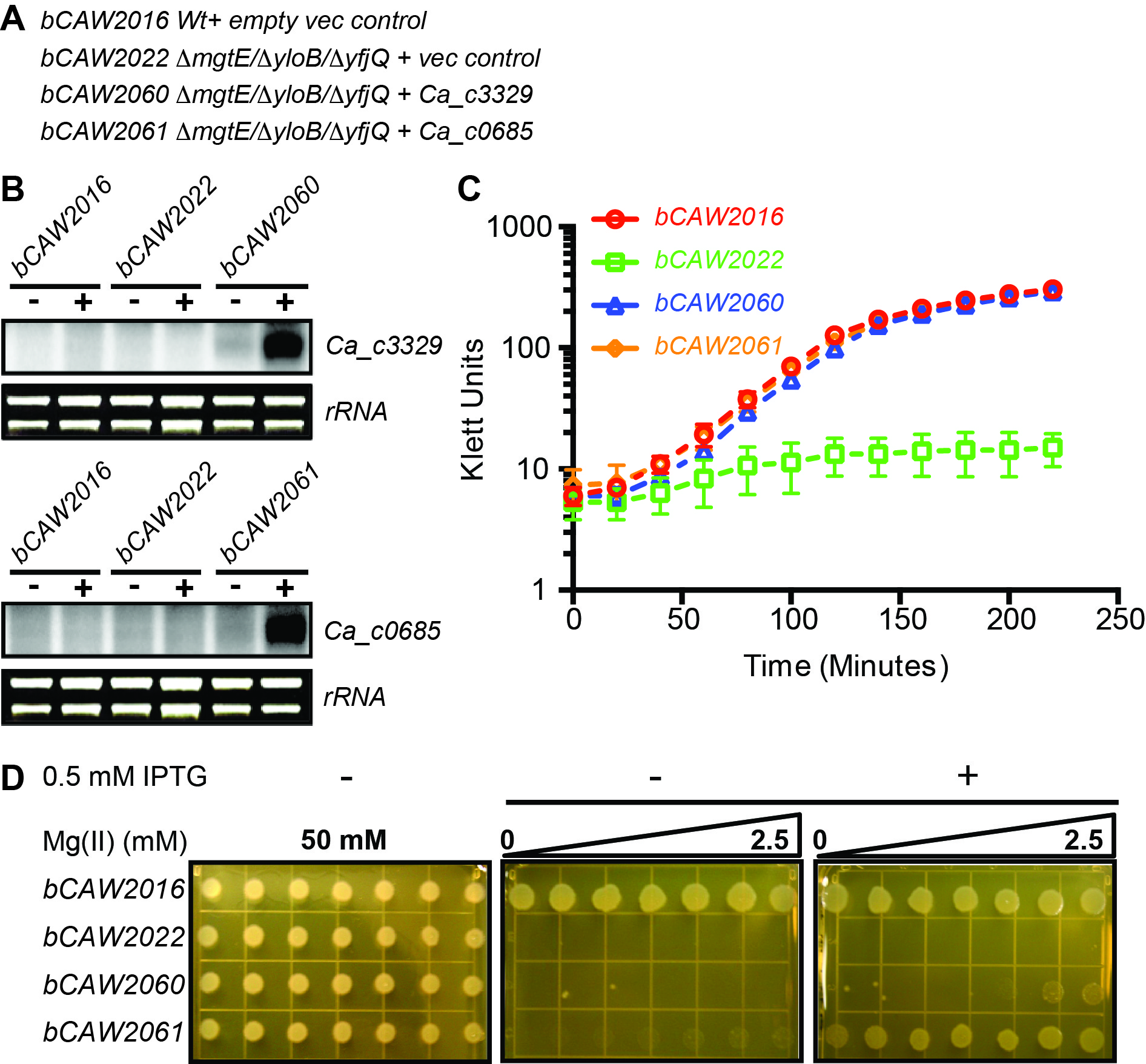

Supplement: Figure S3 — Heterologous expression of Ca_c0685 and Ca_c3329 in a magnesium transport-deficient strain. (A) Genotype legend (Table S1). (B) Strains containing inducible Ca_c0685 and Ca_c3329 analyzed alongside control strains. 0.5 mM IPTG was added to exponentially growing cultures for 1 hr, whereupon 100 µg of total RNA was hybridized with radiolabeled S1 probe DNA respectively. DNA oligonucleotides used for S1 mapping are listed in Table S2. “+” indicates addition of IPTG, whereas “−” indicates the absence of IPTG. Following S1 mapping, the protected DNA probes were analyzed by phosphor imaging. Representative results are presented in this figure. These data indicate that the Ca_c0685 and Ca_c3329 genes are transcribed under these conditions. (C) Growth curves are shown for B. subtilis control strains, including wild-type and a ΔmgtE/ΔyloB/ΔyfjQ triple mutant that is deficient in magnesium transport activity, and transport-deficient strains containing an IPTG-inducible copy of Ca_c0685 or Ca_c3329 integrated into the amyE locus. The resulting strains were cultured in rich medium in the presence of 0.5 mM IPTG and 2.5 mM magnesium. Expression of Ca_c0685 and Ca_c3329 both fully rescued growth in this medium. (D) Also, 3 µL of each of these strains (∼1×10^4^/µL) was spotted onto solid medium containing a gradient of magnesium that ranged from 0 to 2.5 mM magnesium. These plates were incubated for 10 hours at 37°C before they were photographed. These results revealed that Ca_c0685 fully rescued growth of the magnesium-deficient strain whereas Ca_c3329 only rescued growth in the presence of low millimolar magnesium. (JPG) [file pgen.1004429.s003.jpg]

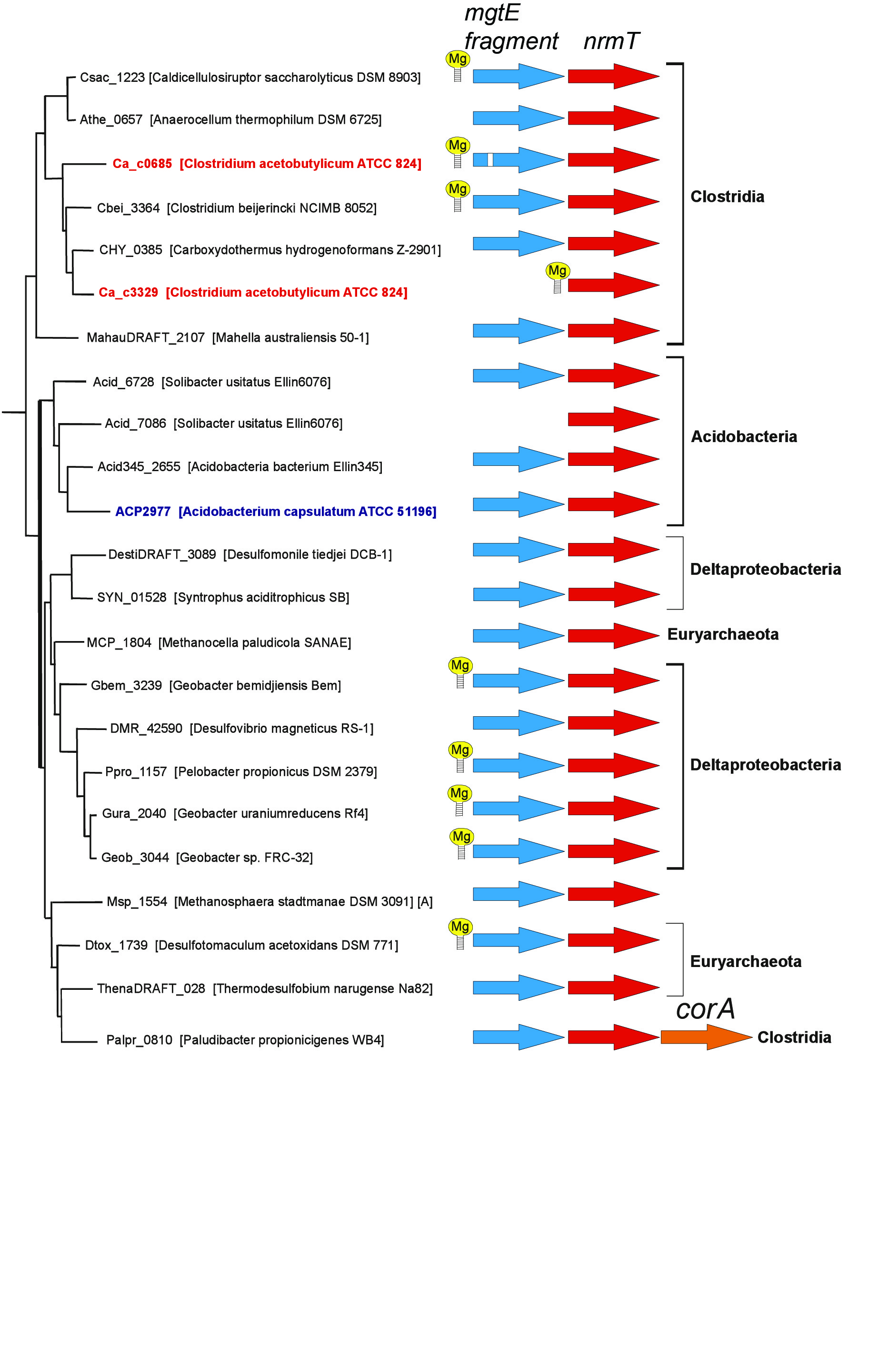

Supplement: Figure S5 — Genome context analysis of MgtE-associated branch of NrmT genes. M-box riboswitches were identified in the promoter regions of mgtE-nramp operons using the Rfam database of RNA motifs. Phylogenetic tree for the group of related proteins using was constructed using the MicrobesOnline genomic database. Experimentally tested transporters from C. acetobutylicum and A. capsulatum and are in red and blue, respectively. (JPG) [file pgen.1004429.s005.jpg]

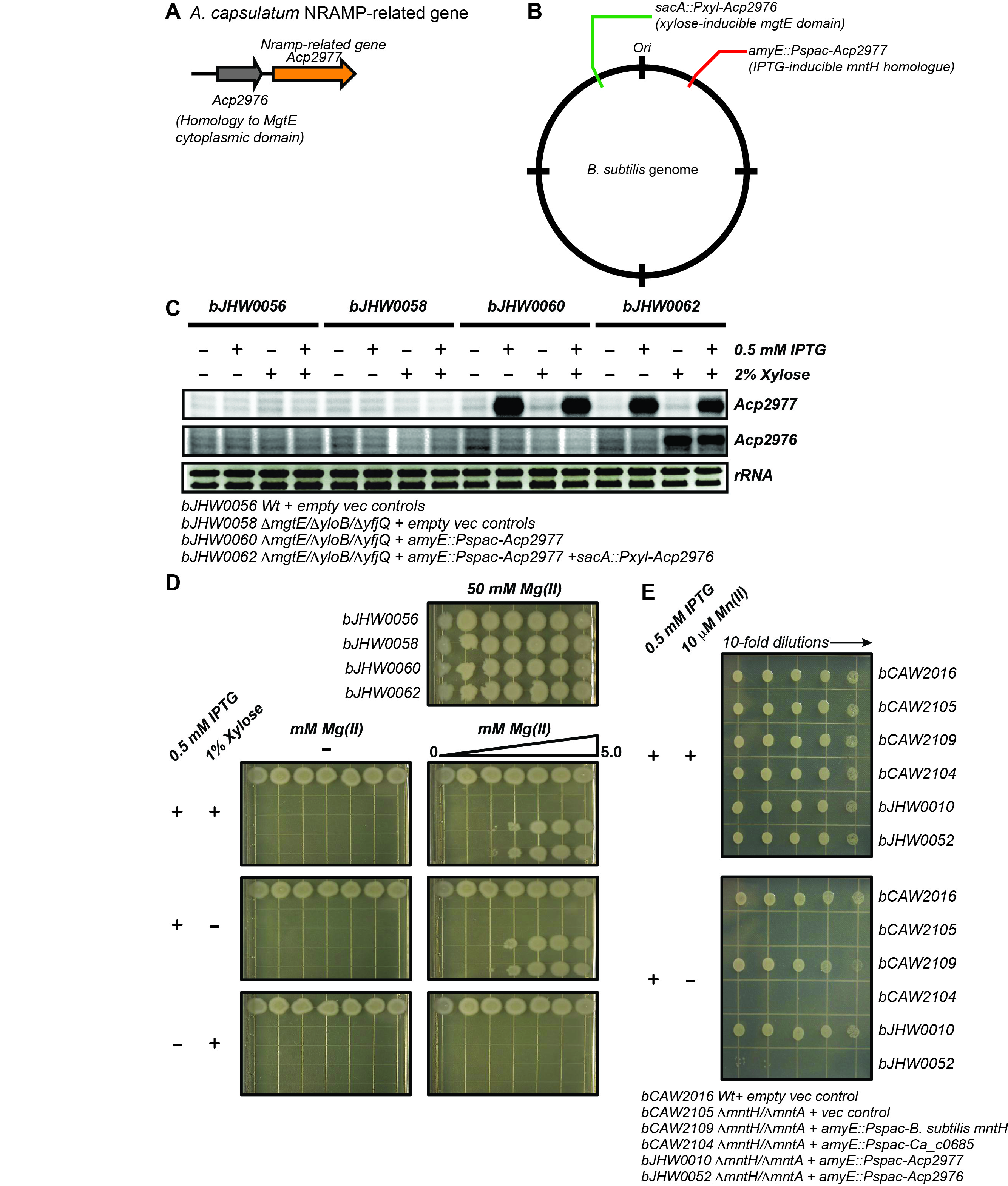

Supplement: Figure S6 — Expression of A. capsulatum ACP2976 and ACP2977. (A) Schematic representation of the gene arrangement of a Nramp-related gene from Acidobacterium capsulatum. This particular Nramp relative (Acp2977) was chosen as it is related to the magnesium-transporting Ca_c0685 gene but lacks an observable magnesium riboswitch. However, it, like the majority of magnesium associated Nramp homologues is located immediately downstream of an open reading frame that appears to encode for a protein that is homologous to the cytoplasmic domain of the magnesium transporter, MgtE (Acp2976). (B) The Acp2977 gene was integrated into the B. subtilis amyE gene while the Acp2976 gene was integrated into the sacA locus under IPTG- and xylose-inducible control, respectively. The background B. subtilis strain also included deletions of three putative magnesium transporters, mgtE, yloB, and yfjQ. (C) Analysis of this and other control strains by S1 mapping showed that the Acp2976 and Acp2977 genes were indeed transcribed when induced by xylose and IPTG, respectively. (D) 3 µl of these strains (∼1×10^4^/µL) was spotted onto solid medium containing a gradient of magnesium from 0 to 5 mM magnesium. These plates were incubated for 10 hours at 37°C before they were photographed. These results revealed that the Nramp homologue, Acp2977, could partially rescue growth of the magnesium-deficient strain whereas Acp2976 alone was unable to rescue growth under these conditions. (E) Strains that included IPTG-inducible copies of either Acp2977 or Acp2976 and that included deletion of manganese transport genes were serially diluted onto solid growth medium that either contained or lacked 10 µM manganese. Several control strains are included in this analysis and are described in the figure. Under these conditions induction of B. subtilis MntH was sufficient for rescue of growth in the absence of added manganese. Similarly, induction of Acp2977 fully rescued growth in the absence of added manganese; therefore, the [file pgen.1004429.s006.jpg]
