# Supplementary material for: Transport of Magnesium by a Bacterial Nramp-Related Gene
Source: PLoS Genet. 2014 Jun 26;10(6):e1004429. doi: 10.1371/journal.pgen.1004429 (PMC4072509; doi:10.1371/journal.pgen.1004429)
Supplement: Figure S4 — Multiple sequence alignment of Nramp transporters. Magnesium-associated branch regulated by M-box riboswitches is in red font. Selected conserved residues that differ in the magnesium-associated genes or are important for manganese/iron uptake in MntH from E. coli [61] are indicated by annotation. (PDF) [file pgen.1004429.s004.pdf]

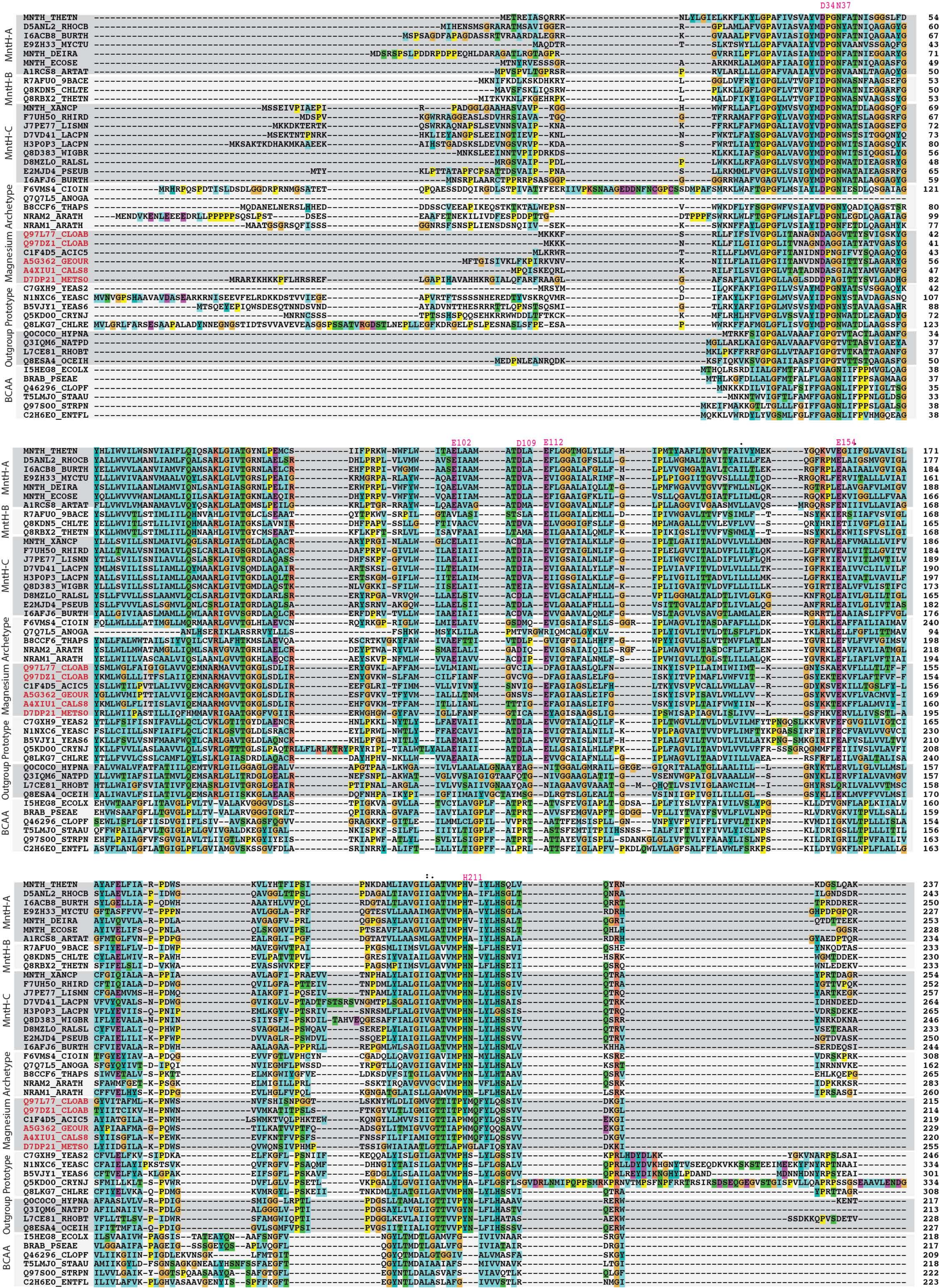

D34N37
E102
D109
E112
E154
H211
MntH-A
MntH-B
MntH-C
Archetype
Magnesium
Prototype
Outgroup
BCAA

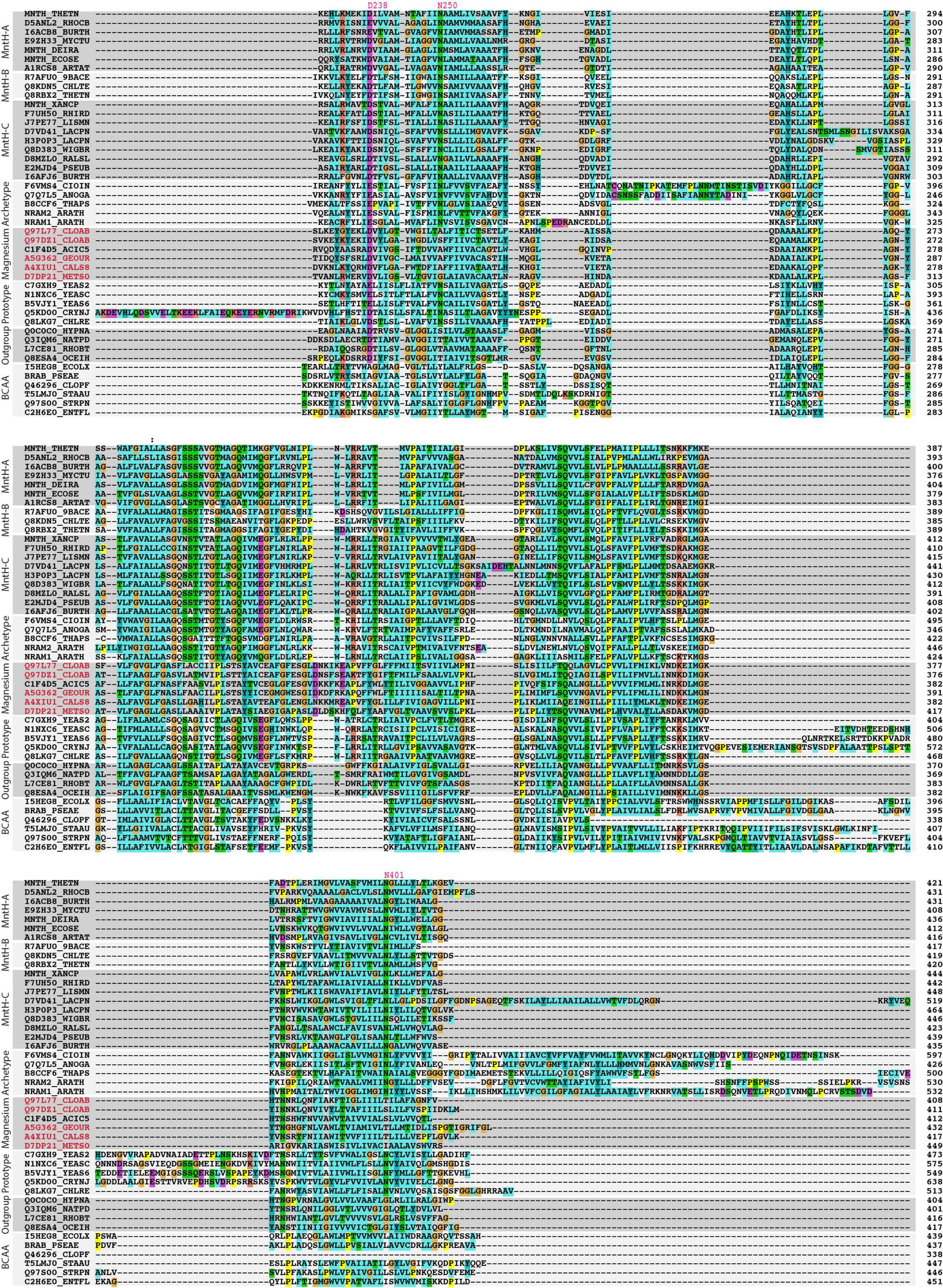
D238
N250
N401
MntH-A
MntH-B
MntH-C
Archetype
Magnesium
Prototype
Outgroup
BCAA
MNTH_THETN
D5ANL2_RHOCB
I6ACB8_BURTH
E9ZH33_MYCTU
MNTH_DEIRA
MNTH_ECOSE
A1RCS8_ARTAT
R7AFU0_9BACE
Q8KDN5_CHLTE
Q8RBX2_THETN
MNTH_XANCP
F7UH50_RHIRD
J7PE77_LISMN
D7VD41_LACPN
H3P0P3_LACPN
Q8D383_WIGBR
D8MZL0_RALSL
E2MJD4_PSEUB
I6AFJ6_BURTH
F6VMS4_CIOIN
Q7Q7L5_ANOGA
B8CCF6_THAPS
NRAM2_ARATH
NRAM1_ARATH
Q97L77_CLOAB
Q97DZ1_CLOAB
C1F4D5_ACIC5
A5G362_GEOUR
A4XIU1_CALS8
D7DP21_METS0
C7GXH9_YEAS2
N1NXC6_YEASC
B5VJY1_YEAS6
Q5KD00_CRYNJ
Q8LKG7_CHLRE
Q0C0C0_HYPNA
Q3IQM6_NATPD
L7CE81_RHOBT
Q8ESA4_OCEIH
I5HEG8_ECOLX
BRAB_PSEAE
Q46296_CLOPF
T5LMJO_STAAU
Q97S00_STRPN
C2H6E0_ENTFL
