# Supplementary material for: Transport of Magnesium by a Bacterial Nramp-Related Gene
Source: PLoS Genet. 2014 Jun 26;10(6):e1004429. doi: 10.1371/journal.pgen.1004429 (PMC4072509; doi:10.1371/journal.pgen.1004429)
Supplement: Table S1 — Bacillus subtilis strains and plasmids used in this study. Shown in this table are all of the strains and relevant plasmids for the experimentation described herein. The construction and characterization of the magnesium transporter deletion strains, including the marker-less ΔmgtE, ΔyloB, and ΔyfjQ deletion strains, are described in detail in a separate publication [57]. Similarly, construction and characterization of markerless deletions of mntABCD, and mntH are also described in this publication [57]. (DOCX) [file pgen.1004429.s007.docx]

Table S1. *Bacillus subtilis* strains and plasmids used in this study.

| **Strain or plasmid** | | **Genotype or description** | **Reference or source** |
| --- | --- | --- | --- |
| ***B. subtilis* strains** | |  |  |
|  | 168 | Parental/ *trpC2* | Bacillus Genetic Stock Center, Columbus Ohio |
|  | bCAW2016 | *B. subtilis* 168 | This study |
|  |  | *amyE::Pspac* (from integration of pHyperspank) (Spec^R^) |  |
|  | bCAW2006 | Markerless deletion of *yloB*, *yfjQ*, and *ykoK* genes using method by Arnaud et al., 2004 (50 mM Mg (II)) | Wakeman et al., 2014 |
|  | bCAW2022 | (bCAW2006) Δ*yloB*, Δ*yfjQ*, Δ*ykoK* | This study |
|  |  | *amyE::Pspac* (from Hyperspank) (Spec^R^) |  |
|  | bCAW2060 | (bCAW2006) Δ*yloB*, Δ*yfjQ*, Δ*ykoK* | This study |
|  |  | *amyE::Pspac*-*ca_c3329* (Spec^R^) |  |
|  | bCAW2061 | (bCAW2006) Δ*yloB*, Δ*yfjQ*, Δ*ykoK* | This study |
|  |  | *amyE::Pspac*-*ca_c0685* (Spec^R^) |  |
|  | bCAW2073 | (bCAW2006) Δ*yloB*, Δ*yfjQ*, Δ*ykoK* | This study |
|  |  | *amyE::Pspac*-*mntA-D* (Spec^R^) |  |
|  | bCAW2076 | (bCAW2006) Δ*yloB*, Δ*yfjQ*, Δ*ykoK* | This study |
|  |  | *amyE::Pspac*-*mntH* (Spec^R^) |  |
|  | bCAW2069 | 168 Δ*mntH* (markerless) | This study |
|  | bCAW2087 | 168 Δ*mntH* Δ*mntA* (markerless deletions) (Requires >10 μM Mn (II)) | Wakeman et al., 2014 |
|  | bCAW2105 | 168 Δ*mntH* Δ*mntA* (markerless deletions) | This study |
|  |  | *amyE::Pspac* (from pHyperspank) (Spec^R^) |  |
|  | bCAW2104 | 168 Δ*mntH* Δ*mntA* (markerless deletions) | This study |
|  |  | *amyE::Pspac*-*ca_c0685* (Spec^R^) |  |
|  | bCAW2109 | 168 Δ*mntH* Δ*mntA* (markerless deletions) | This study |
|  |  | *amyE::Pspac*-*mntH* (Spec^R^) |  |
|  | bCAW2114 | 168 Δ*mntH* Δ*mntA* (markerless deletions) | This study |
|  |  | *amyE::Pspac*-*ca_c3329* (Spec^R^) |  |
|  | PY79 | Prototrophic *B. subtilis* | Bacillus Genetic Stock Center |
|  | bJHW0001 | PY79 *amyE*::*PrpsD-yfp* (Chloramphenicol^R^) | This study |
|  | bJHW0002 | PY79 *amyE*::*PrpsD-ca_c3329Mbox-yfp* (Chloramphenicol^R^) | This study |
|  | bJHW0003 | PY79 *amyE*::*PrpsD-ca_c0685Mbox-yfp* (Chloramphenicol^R^) | This study |
|  | bJHW0009 | (bCAW2006) Δ*yloB*, Δ*yfjQ*, Δ*ykoK* | This study |
|  |  |  |  |
|  |  | *amyE::pSpac-Acp2977* (Chloramphenicol^R^) |  |
|  | bJHW0056 | *B. subtilis* 168 | This study |
|  |  | *amyE::Pspac* (from integration of pHyperspank) |  |
|  |  | *sacA::pXylA* (from integration of pIR1127) |  |
|  |  | (Chloramphenicol^R^ Spec^R^) |  |
|  | bJHW0010 | 168 Δ*mntH* Δ*mntA* (markerless deletions) (Requires >10 μM Mn (II)) | This study |
|  |  | *amyE::Pspac-Acp2977* |  |
|  |  | (Chloramphenicol^R^ Spec^R^) |  |
|  | bJHW0052 | 168 Δ*mntH* Δ*mntA* (markerless deletions) (Requires >10 μM Mn (II)) | This study |
|  |  | *amyE::Pspac-Acp2976* |  |
|  |  | (Chloramphenicol^R^ Spec^R^) |  |
|  | bJHW0058 | Δ*yloB*, Δ*yfjQ*, Δ*ykoK* | This study |
|  |  | *amyE::Pspac* (from integration of pHyperspank) |  |
|  |  | *sacA::pXylA* (from integration of pIR1127) |  |
|  |  | (Chloramphenicol^R^ Spec^R^) |  |
|  | bJHW0060 | Δ*yloB*, Δ*yfjQ*, Δ*ykoK* | This study |
|  |  | *amyE::Pspac-Acp2977* |  |
|  |  | *sacA::pXylA* (from integration of pIR1127) |  |
|  |  | (Chloramphenicol^R^ Spec^R^) |  |
|  | bJHW0062 | Δ*yloB*, Δ*yfjQ*, Δ*ykoK* | This study |
|  |  | *amyE::Pspac-Acp2977* |  |
|  |  | *sacA::pXylA-Acp2976* |  |
|  |  | (Chloramphenicol^R^ Spec^R^) |  |
| ***E. coli* strains** | |  |  |
|  | DH5α | *F- endA1 glnV44 thi-1 recA1 relA1 gyrA96 deoR nupG Φ80dlacZΔM15 Δ(lacZYA-argF)U169, hsdR17(rK- mK+), λ–* | Lab stock |
| **Plasmids** | |  |  |
|  | pDG1662 | Integrates by double homologous recombination into *amyE* Integrants are Cm^R^Sp^S^. | Gue'rout-Fleury, A-M *et al*., 1996 |
|  | pMAD | Used for creating markerless mutations in Gram-positive bacteria. | Arnaud, M *et al*., 2004 |
|  | pHyperspank | *amyE* integration vector containing a promoter that is inducible addition of isopropyl β-D-1-thiogalactopyranoside (IPTG). | Van Ooij C and Losick R. 2003 |
|  | pIR1127 | Altered version of pSac-CM to contain a xylose-inducible promoter near to a xylR gene (which derives from pSweetIII) | Unpublished data |

References:

Arnaud M, Chastanet A, Débarbouillé M. 2004. New vector for efficient allelic replacement in naturally nontransformable, low-GC-content, Gram-positive bacteria. *Appl Environ Microbiol* 70:6887-91.

Gue-rout-Fleury AM, Frandsen N, Stragier P. 1996. Plasmids for ectopic integration in *Bacillus subtilis*. *Gene* 180:57-61.

Van Ooij C, Losick R. 2003. Subcellular localization of a small sporulation protein in *Bacillus subtilis. J Bacteriol* 185:1391-8.

Wakeman CA, Goodson JR, Zacharia VM, Winkler WC (2014) An Assessment of the Requirements for Magnesium Transporters in Bacillus subtilis. *J Bacteriol*. doi:10.1128/JB.01238-13.
