# Supplementary material for: Transport of Magnesium by a Bacterial Nramp-Related Gene
Source: PLoS Genet. 2014 Jun 26;10(6):e1004429. doi: 10.1371/journal.pgen.1004429 (PMC4072509; doi:10.1371/journal.pgen.1004429)
Supplement: Table S2 — The DNA oligonucleotides that were used in this study are listed and briefly described herein. (DOCX) [file pgen.1004429.s008.docx]

Table S2 Oligonucleotides used in this study

| Function and primer name | | Primer sequence (5’ to 3’) | Description |
| --- | --- | --- | --- |
| Mutant construction | |  |  |
|  | CAW273 | CTCGTTGGATCCGGCTGCTGACCCAGCCGG | Forward oligo for amplification of *mntABCD* flanking region 1 |
|  | CAW274 | gctgagtccctgcaggccttagaggacatcctccattttccc | Reverse oligo for amplification of *mntABCD* flanking region 1 |
|  | CAW275 | gggaaaatggaggatgtcctctaaggcctgcagggactcagc | Forward oligo for amplification of *mntABCD* flanking region 2 |
|  | CAW276 | tccggagaattcgggcacaagaccgagaatcg | Reverse oligo for amplification of *mntABCD* flanking region 2 |
|  | CAW279 | tctcccggatccaaagccgtccaagatgcg | Forward oligo for amplification of *mntH* flanking region 1 |
|  | CAW280 | cagtacgagagcggtcgtttacagtcccagagccgctcc | Reverse oligo for amplification of *mntH* flanking region 1 |
|  | CAW281 | ggagcggctctgggactgtaaacgaccgctctcgtactg | Forward oligo for amplification of *mntH* flanking region 2 |
|  | CAW282 | Tttccctgtcgactatgggcagcaacagaac | Reverse oligo for amplification of *mntH* flanking region 2 |
| Complementation Plasmids | |  |  |
|  | CAW186 | aaaaattgtcgacgtgggaaagctgcttcaattg | Forward oligo for amplification of *Ca_c3329* |
|  | CAW187 | tttataggcatgcaccccttatcggaaaccg | Reverse oligo for amplification of *Ca_c3329* |
|  | CAW188 | tttaattgtcgacgtactcctaccagcgtgg | Forward oligo for amplification of *Ca_c0685* |
|  | CAW189 | atataaagcatgctctatacaaaatttccagc | Reverse oligo for amplification of *Ca_c0685* |
|  | JHW0123 | CGCTGAAGTCGACAAGGAGGAAAG | Forward oligo for amplification of *Acp2977* for subcloning into pHyperspank |
|  | JHW0124 | CTTTGACGGCTAGCCAAAAAACCCC | Reverse oligo for amplification of *Acp2977* for subcloning into pHyperspank |
|  | JHW0070 | CGTATAGGAGACCTATAGTGTCGACGGGGA TCCAAG | Forward oligo for amplification of *Acp2976* for subcloning into pHyperspank |
|  | JHW0071 | GGAGACGTATATGGTCTTCGCTAGCGGCCGCC | Reverse oligo for amplification of *Acp2976* for subcloning into pHyperspank |
|  | JHW0072 | CCTATAGTGTCTTCGCGGCCGCAAGGAGGA AAG | Forward oligo for amplification of *Acp2977* for subcloning into pIR1127 |
|  | JHW0073 | GGTCTTCTTTTGCGGATCCCAAAAAACCCCTC | Reverse oligo for amplification of *Acp2977* for subcloning into pIR1127 |
| YFP fusion | |  |  |
|  | JHW0049 | CTTCAAAACAAATAAATTATATAATGACCTTTGTGTGAATAGTAACATGATGTCACCTCC | Forward oligo for amplification of *Ca_c3329* M-box and transcriptional fusion to *yfp* gene |
|  | JHW0050 | TCTTCACCTTTGCTCATAATGTGACTTTCCTCCTTAATCAGTTTTCCCTCCTCAATGCAG | Reverse oligo for amplification of *Ca_c3329* M-box and transcriptional fusion to *yfp* gene |
|  | JHW0051 | TTCAAAACAAATAAATTATATAATGACCTTTGTGTGAAAAGTTTTAGTTAGGTGAGGCTT | Forward oligo for amplification of *Ca_c0685* M-box and transcriptional fusion to *yfp* gene |
|  | JHW0052 | TCTTCACCTTTGCTCATAATGTGACTTTCCTCCTTAATTTAGCTTGAAGAGAAAGATTGG | Reverse oligo for amplification of *Ca_c0685* M-box and transcriptional fusion to *yfp* gene |
| Oligos used for S1 mapping analysis | |  |  |
|  | JHW0001 | CAGGGCGGATCCCAACCTATGC | *Ca_c3329* S1 Forward oligo/BamHI |
|  | JHW0002 | CCCTTATTAAATCTGATAGTCC | *Ca_c3329* S1 Reverse oligo |
|  | JHW0003 | GCAGGAGGGATCCCTACCTATTC | *Ca_c0685* S1 Forward oligo /BamHI |
|  | JHW0004 | CAACTTTACGCCGTACCTTTCTC | *Ca_c0685* S1 Reverse oligo |
|  | JHW0013 | CAGCCGGGGATCCCGCTGATCAG | *MntA* S1 Forward oligo /BamHI |
|  | JHW0014 | GGTGCGGATCAACCCCCGGTCCC | *MntA* S1 Reverse oligo |
|  | JHW0018 | GACGGACACGCGTTTGTGCGTGTCT | *YciA* Forward oligo S1 for Zn |
|  | JHW0019 | CCACTGGTTTCTCGCCTTTGATCGG | *YciA* Reverse oligo S1 for Zn |
|  | JHW0020 | CCCTTCTCGTTTGGACCGGCTGCTG | *MntA* Forward oligo S1 for Mn |
|  | JHW0021 | CCGGCTGAATCCGTTCCGCATCCCG | *MntA* Reverse oligo s1 for Mn |
|  | JHW0022 | GGTTGACAAGAAACCGGGATGGTC | *MntH* Forward oligo S1 for Mn |
|  | JHW0023 | GCCGAGAAAAGGGAGCAGCCCTCTG | *MntH* Reverse oligo S1 for Mn |
|  | JHW0024 | GGTTCAAAACATGACCTAACTCGAACTC | *MgtE* Forward oligo S1 for Mg |
|  | JHW0025 | GCTCCCCGATCATATCAGTGATATCGTC | *MgtE* Reverse oligo S1 for Mg |
|  | JHW0026 | CACCCTCTCATTCTTCCGCGATTCG | *MgtA* Forward oligo S1 for Mg |
|  | JHW0027 | CGTTTGGTCCATGCTTGTCAAGGCG | *MgtA* Reverse oligo S1 for Mg |
|  | JHW0028 | GGGCTGAGACATACTCAGCCTTGCC | *DhbA* Forward oligo S1 for Fe |
|  | JHW0029 | CGCAACAGCTTCGCCTATTCCTTGG | *DhbA* Reverse oligo S1 for Fe |
|  | JHW0030 | GACGGGGAACAGGCCATTTCTGAG | *CopZ* Forward oligo S1 for Cu |
|  | JHW0031 | CGCTTCCAGATTGACATGAACGGC | *CopZ* Reverse oligo S1 for Cu |
|  | JHW0032 | CACCGGCGTTGTGCCAATCGAGGTTGAG | YFP Forward oligo S1 |
|  | JHW0033 | TCTTGGACATAGCCTTCCGGCATGGCAG | YFP Reverse oligo S1 |
|  | JHW0083 | CGCAATACGGATCCTCGCTGCTCTGGAC | *Acp2977* S1 BamHI |
|  | JHW0084 | GCAGGCCAAATTCTTCGCGGATGAGGTC | *Acp2977* S1 |
|  | JHW0085 | CGACCCTGACTAGGATCCTTGGCACTCCGGTGGC | *Acp2976* S1 BamHI |
|  | JHW0086 | GCAAGTCTATGGCGGAGACCACCTGCAGCCCATCG | *Acp2976* S1 |
